# EGFR Targeted Liposomal PROTAC Assisted With Epigenetic Regulation as an Efficient Strategy for Osimertinib‐Resistant Lung Cancer Therapy

**DOI:** 10.1002/advs.202510197

**Published:** 2025-08-21

**Authors:** Dongyuan Wang, Yajing Liu, Ying Chen, Chuan Dai, Wenzhu Hu, Jinyan Han, Zigang Li, Feng Yin, Yu Zhang, Chen Shi

**Affiliations:** ^1^ Department of Pharmacy Union Hospital Tongji Medical College Huazhong University of Science and Technology Wuhan 430022 China; ^2^ Hubei Province Clinical Research Center for Precision Medicine for Critical Illness Wuhan 430022 China; ^3^ State Key Laboratory of Chemical Oncogenomic School of Chemical Biology and Biotechnology Peking University Shenzhen Graduate School Shenzhen 518055 China; ^4^ Pingshan Translational Medicine Center Shenzhen Bay Laboratory Shenzhen 518118 China; ^5^ Department of Nuclear Medicine Union Hospital Tongji Medical College Huazhong University of Science and Technology Wuhan 430022 China

**Keywords:** epigenetic regulation, HDAC, liposomal PROTAC, lung cancer, osimertinib resistance

## Abstract

Osimertinib resistance is a global problem for NSCLC patients mediated by new EGFR mutations or bypass mechanisms. Proteolysis targeting chimeras (PROTACs) have been utilized to overcome drug resistance by degrading mutant EGFR, but most are restricted to their poor cell permeability and insufficient tumor‐targeting ability. Meanwhile, these PROTACs has little effect on bypass resistance mechanisms. In this study, a versatile split‐and‐mix liposomal PROTAC is developed for EGFR degradation based on liposome self‐assembly containing DSPE‐PEG2000‐E3 ligand and DSPE‐PEG2000‐EGFR ligand. Unlike traditional PROTACs, this platform can achieve efficient EGFR degradation via both E3‐dependent mechanisms and the lysosome‐autophagy pathway. To further increase its sensitivity to osimertinib‐resistant lung cancer cells, the liposomal PROTAC is encapsulated with class I HDAC inhibitor MS‐275 (GM‐protac) by both blocking the EGFR‐dependent pathways and bypass resistant mechanisms. GM‐protac shows selective toxicity on gefitinib‐resistant and osimertinib‐resistant lung cancer cells. The mechanism analysis reveals that GM‐protac can influence the BIM‐associated apoptosis pathway, c‐Met, PD‐L1, HER‐2, NF‐κB or PI3K‐AKT signaling pathway, etc. Meanwhile, they displayed obvious tumor inhibition with negligible toxicity in both osimertinib and gefitinib‐resistant lung cancer animal models. This work provides an alternative option for osimertinib‐resistant lung cancer therapy.

## Graphical abstract

In this study, an EGFR lipoSM PROTACs (GM‐protac), which can achieve efficient EGFR degradation and HDACi delivery, is developed. It is found that GM‐protac can both block EGFR‐dependent pathways and bypass the HDAC signaling pathway. Meanwhile, both display obvious tumor inhibition with negligible toxicity in EGFR‐TKI‐resistant lung cancer animal models. This work provides an alternative option for osimertinib‐resistant lung cancer therapy.

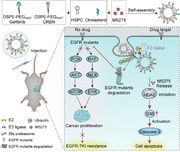

## 1 Introduction

Non‐small cell lung cancer (NSCLC) is the most frequent histological subtype of lung cancer, accounting for ≈85% of all lung cancer patients.^[^ ^1^, ^2^, ^3^, ^4^ ^]^ Epidermal growth factor receptor (EGFR), plays a crucial role in regulating lung cancer cell proliferation, migration, and survival, and is considered as an important biomarker in non‐small cell lung cancer (NSCLC).^[^ ^5^, ^6^, ^7^ ^]^ Despite patients deriving significant benefit from various EGFR‐tyrosine kinase inhibitors (EGFR‐TKIs), they will inevitably develop acquired resistance mediated by EGFR‐dependent or bypass resistance mechanisms.^[^ ^6^, ^8^, ^9^, ^10^ ^]^ The approved EGFR‐TKIs include the first generation TKIs such as gefitinib, icotinib, which target EGFR exon 19 deletion or exon 21 L858R mutation; the second generation TKIs such as afatinib, dacomitinib, which target the same EGFR mutations as the first generation TKIs, but had more potent therapeutic effect and better safety due to the covalent inhibition; the third generation TKIs such as osimertinib and almonertinib, which target EGFR T790M mutation, which is resistant to the first or second‐generation TKIs.^[^ ^6^ ^]^ Despite the new mutations occurred, such as C797S mutants, which are resistant to the third‐generation TKIs, there is no drug approved for it. A deeper understanding of tumor heterogeneity and the identification of specific resistance mechanisms might help to overcome EGFR‐TKI resistance. The complexity of drug resistance requires the combination of EGFR‐TKIs with different drugs associated with bypass signaling pathway, which have been widely investigated under preclinical and clinical applications.^[^ ^11^, ^12^, ^13^, ^14^ ^]^

The frequent drug resistance caused by EGFR mutations makes the new generation of EGFR inhibitors helpless in the treatment of NSCLC. Proteolysis targeting chimeras (PROTACs), consisting of targeted protein ligand, E3 ligand, and a linker to connect the two ligands, have emerged as a new and promising therapeutic modality that induces targeted protein degradation by ubiquitin‐proteasome machinery.^[^ ^15^, ^16^ ^]^ PROTACs provide many advantages over traditional inhibitors, such as targeting undruggable targets, overcoming drug resistance, and reducing drug doses in the clinic.^[^ ^17^, ^18^, ^19^, ^20^ ^]^ In recent years, several EGFR targeting PROTACs have been reported, which degrade EGFR L858R/T790M, Del19/T790M double mutants, or EGFR L858R/T790M/C797S mutants and exhibit potent anti‐proliferation activities.^[^ ^17^, ^21^, ^22^, ^23^, ^24^ ^]^ Despite these advancements, PROTACs are still limited by their poor membrane permeability, low water solubility, and systemic toxicity.^[^ ^25^, ^26^, ^27^ ^]^

To improve the therapeutic efficacy of PROTACs, various tumor‐targeting strategies have been developed including: 1) conjugation with tumor‐homing moieties, such as aptamer‐PROTAC,^[^ ^28^ ^]^ antibody‐PROTACs etc.;^[^ ^29^ ^]^ 2) stimuli‐activatable prodrugs with PROTACs inspired by enzymes, and light etc.;^[^ ^30^ ^]^ 3) nano‐sized drug delivery systems to deliver PROTACs via their enhanced permeability and retention (EPR) effect.^[^ ^31^ ^]^ However, all these strategies couldn't circumvent the meticulous design, time‐consuming screening of small PROTACs with complicated steps, and large testing agent consumption. To address these challenges, we previously proposed a split‐and‐mix PROTAC nanoplatform (SM‐PROTAC) with facile screening, programmable ligand ratios, self‐optimized biomolecule spatial recognition.^[^ ^32^, ^33^, ^34^, ^35^ ^]^ As a universal delivery system, split‐and‐mix liposome PROTAC (LipoSM‐PROTAC) achieved therapeutic efficacy with a low concentration, which provides opportunities for clinical translational potential.^[^ ^33^, ^34^, ^35^ ^]^

Besides degrading EGFR mutants, it is crucial to synergistically disrupt bypass‐resistant mechanisms to overcome drug resistance.^[^ ^36^, ^37^ ^]^ Histone deacetylases (HDACs) are a family of enzymes that deacetylate numerous histone or non‐histone substrates that govern a wide array of biological processes, including cancer initiation and progression.^[^ ^38^, ^39^, ^40^ ^]^ Many studies found that EGFR‐TKI resistance could be circumvented by the combination with HDAC inhibition.^[^ ^41^, ^42^, ^43^ ^]^ For example, Yano et al revealed HDAC inhibition can epigenetically restore BIM function and death sensitivity of EGFR‐TKI in cases of EGFR‐mutant NSCLC.^[^ ^44^ ^]^ Later they found that histone deacetylase 3 (HDAC3) inhibition could overcome osimertinib resistance in EGFR‐mutant lung cancer.^[^ ^45^ ^]^ Recently, a novel EGFR and HDAC dual inhibitor was discovered, which exhibited powerful and selective antiproliferative activity against EGFR‐mutant lung cancer compared to wild‐type lung cancer.^[^ ^46^ ^]^ The key mechanisms of HDACi that overcome EGFR‐TKI resistance include inducing oxidative stress‐dependent apoptosis, down‐regulation of the c‐Myc‐regulated nuclear factor erythroid 2‐related factor 2 (NRF2) transcription factor, and the up‐regulation of the NRF2 repressor Kelch‐like ECH‐associated protein 1 regulator (KEAP1), which are dysfunctional in NSCLC and involved in EGFR‐TKI resistance.^[^ ^47^ ^]^ Studies also revealed that inhibition of HDAC1 induced protein phosphatase DUSP1 upregulation to restore gefitinib sensitivity by inhibiting EGFR signaling and inducing apoptosis.^[^ ^48^ ^]^ Meanwhile, the combination of EGFR‐TKI and HDACi could dramatically down‐regulate the expression of several crucial EGFR‐TKI resistance‐related receptor tyrosine kinases, such as HER‐2, c‐Met, IGF1R, and AXL.^[^ ^49^ ^]^ All these studies indicated that the combination of EGFR‐TKI and HDAC inhibitors could be an alternative strategy to overcome EGFR‐TKI resistance in NSCLC.

**Scheme 1:**
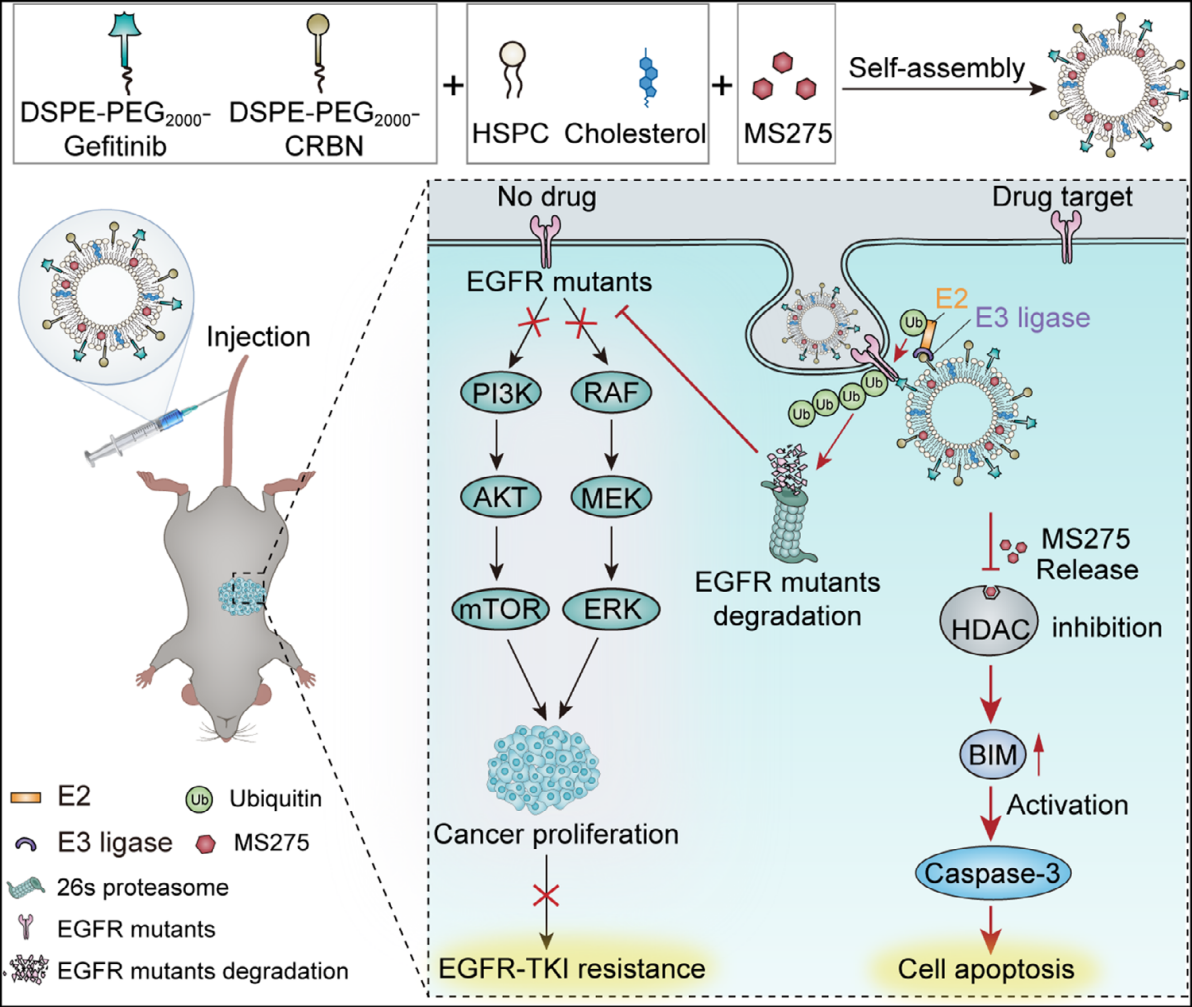
The design of EGFR lipoSM PROTAC in combination with HDACi to overcome EGFR‐TKI resistance in lung cancer cells.

Due to the complexity of drug‐resistant mechanisms, simultaneous inactivation of EGFR mutations and bypass signaling pathways could be a promising strategy to overcome osimertinib resistance in NSCLC. The split‐and‐mix PROTAC platform can not only achieve facile screening of effective EGFR PROTACs, but also act as a vector for HDAC inhibitor delivery. In this study, we constructed an EGFR lipoSM PROTAC encapsulating class I HDAC inhibitor MS‐275 (GM‐protac) for osimertinib‐resistant lung cancer therapy. This system showed potent EGFR degradation at low concentration via both E3‐dependent mechanisms and the lysosome‐autophagy pathway. Besides, GM‐protac showed selective toxicity toward osimertinib‐resistant, almonertinib resistant or gefitinib‐resistant cancer cells due to potent HDAC inhibition. Meanwhile, they displayed obvious tumor inhibition with negligible toxicity in both osimertinib and gefitinib‐resistant lung cancer animal models. Our work provided a promising therapeutic option for overcoming osimertinib resistance in NSCLC (**Scheme** **1**).

## 2 Results

### 2.1 Preparation and Characterization of LipoSM‐PROTAC

**Figure 1:**
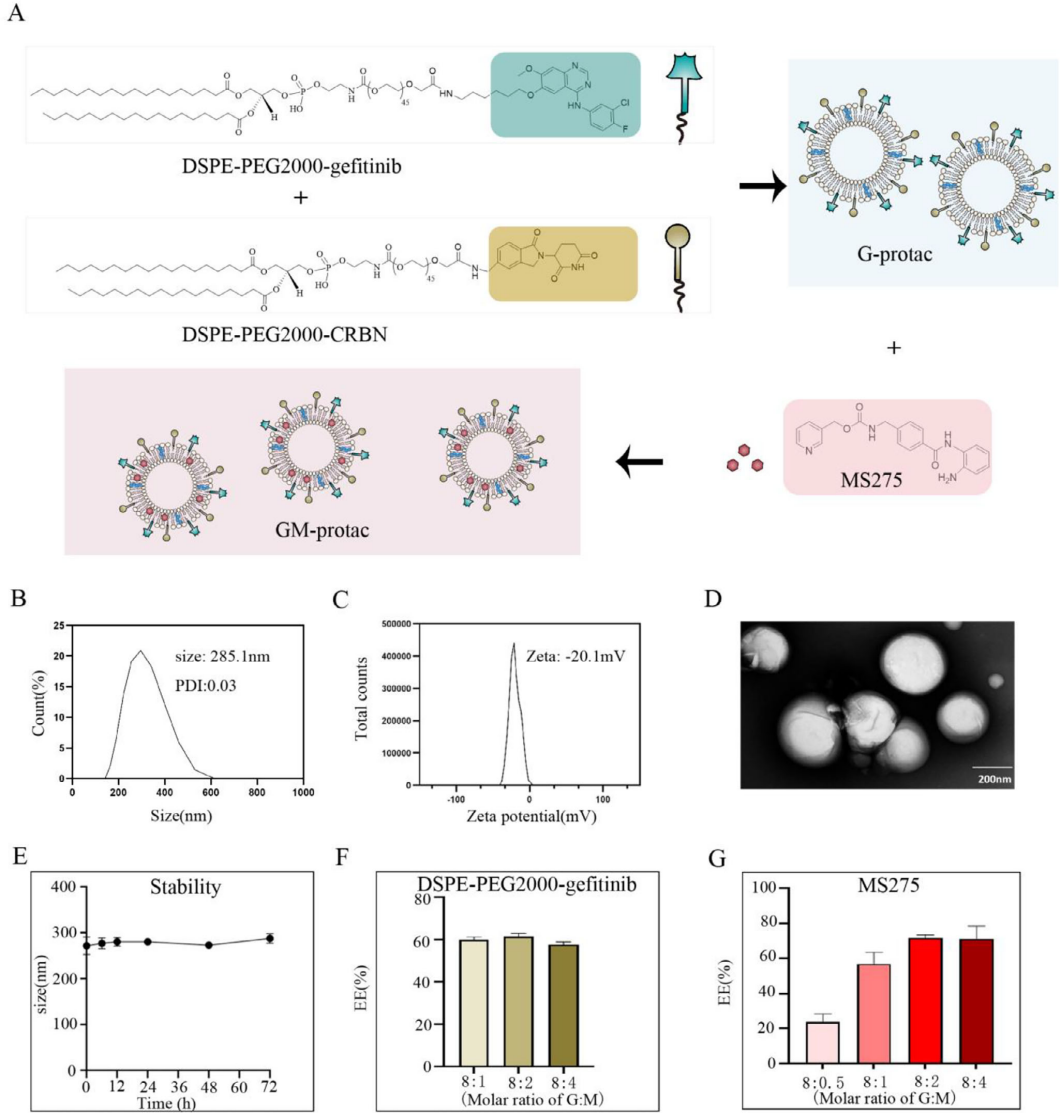
A) Chemical structure of DSPE‐PEG2000‐gefitinib, DSPE‐PEG2000‐CRBN, and MS‐275, and the formation of lipoSM protac containing MS‐275 (GM‐protac). B) Particle size distribution of G‐protac. C) Zeta potential distribution of G‐protac. D) TEM image of G‐protac. Scale bar = 200 nm. E) The stability of G‐protac in PBS. F) Encapsulation efficiency of DSPE‐PEG2000‐gefitinib in G‐protac, the molar ratios DSPE‐PEG2000‐gefitinib/DSPE‐PEG2000‐CRBN include 8:1, 8:2, and 8:4. G) Encapsulation efficiency of MS‐275 in GM‐protac, the molar ratio of DSPE‐PEG2000‐gefitinib/MS‐275 includes 8:0.5, 8:1, 8:2, 8:4. Error bars represent SEMs of at least three independent measurements.

Gefitinib, a first‐generation EGFR‐TKI, has been used as the first‐line treatment for EGFR‐mutant NSCLC. Herein, we used gefitinib as a showcase for EGFR recruiting.^[^ ^50^, ^51^ ^]^ We chose a CRBN E3 ligase recruiting element, which can regulate the intracellular ubiquitin−proteasome system to degrade EGFR mutants, shown in **Figure** **1**A.^[^ ^52^ ^]^ To construct EGFR LipoSM PROTAC, DSPE‐PEG2000‐gefitinib and DSPE‐PEG2000‐CRBN were synthesized shown in Supporting Information, as previously reported.^[^ ^33^ ^]^ and characterized by NMR, ESI‐MS or high‐resolution mass spectrometry (HRMS) shown in Figures S1–S7 (Supporting Information). Then G‐protac (without MS‐275), GM‐protac (containing MS‐275) were prepared using the thin‐film hydration and extrusion method as previously reported.^[^ ^33^ ^]^ and shown in Figure 1A.

As for the preparation of G‐proac, the particle size was around 285 nm with a narrow polydispersity index (PDI) and a negative surface charge (Figure 1B,C). Then the morphology of G‐protac was tested by transmission electron microscope, shown in Figure 1D. We also found G‐protac had good stability even under 72 h with the incubation with PBS, shown in Figure 1E. As gefitinib and MS‐275 play key roles in cancer therapy, we tested their concentration for the encapsulation rate experiment, and the regression coefficient of DSPE‐PEG2000‐gefitinib standard curve is *R* ^2^ = 0.9998, and the regression coefficient of MS‐275 standard curve is *R* ^2^ = 0.9997 shown in Figure S9 (Supporting Information). When the molar ratios of HSPC/Cholesterol/DSPE‐PEG2000‐gefitinib/DSPE‐PEG2000‐CRBN changed from 50:40:8:4 to 50:40:8:2 to 50: 40: 8:1, the encapsulation efficiency did not change a lot, with an average of 60%, shown in Figure 1F. Considering the following degradation efficiency influenced by different molar ratios of DSPE‐PEG2000‐gefitinib/DSPE‐PEG2000‐CRBN as previously reported,^[^ ^33^ ^]^ we chose the ratios of HSPC/Cholesterol/DSPE‐PEG2000‐gefitinib/DSPE‐PEG2000‐CRBN with 50:40:8:1 for the following study.

As for the preparation of GM‐protac, we kept the molar ratios of these components in G‐protac, and increased the molar ratio of DSPE‐PEG2000‐gefitinib: MS‐275. When the ratio of MS‐275: DSPE‐PEG2000‐gefitinib increased to 4:8 or 2:8, the encapsulation efficiency didn't change. Thus the optimized molar ratio of MS‐275: DSPE‐PEG2000‐gefitinib was 4:8 shown in Figure 1G. We also tested the particle size, zeta potential, and morphology of GM‐protac shown as Figure S9 (Supporting Information), which is similar to G‐protac, indicating the encapsulating MS‐275 did not influence the properties of LipoSM protac. To sum up, the optimized molar ratio of HSPC/Cholesterol/DSPE‐PEG2000‐gefitinib/DSPE‐PEG2000‐CRBN/MS‐275 in the lipoSM‐PROTAC was 50:40:8:2:4. We next used this prescription for further in vitro or in vivo assays according to these experimental conditions.

### 2.2 The Synergistic Effect of HDAC Inhibition and EGFR Degradation in Different Lung Cancer Cells

**Figure 2:**
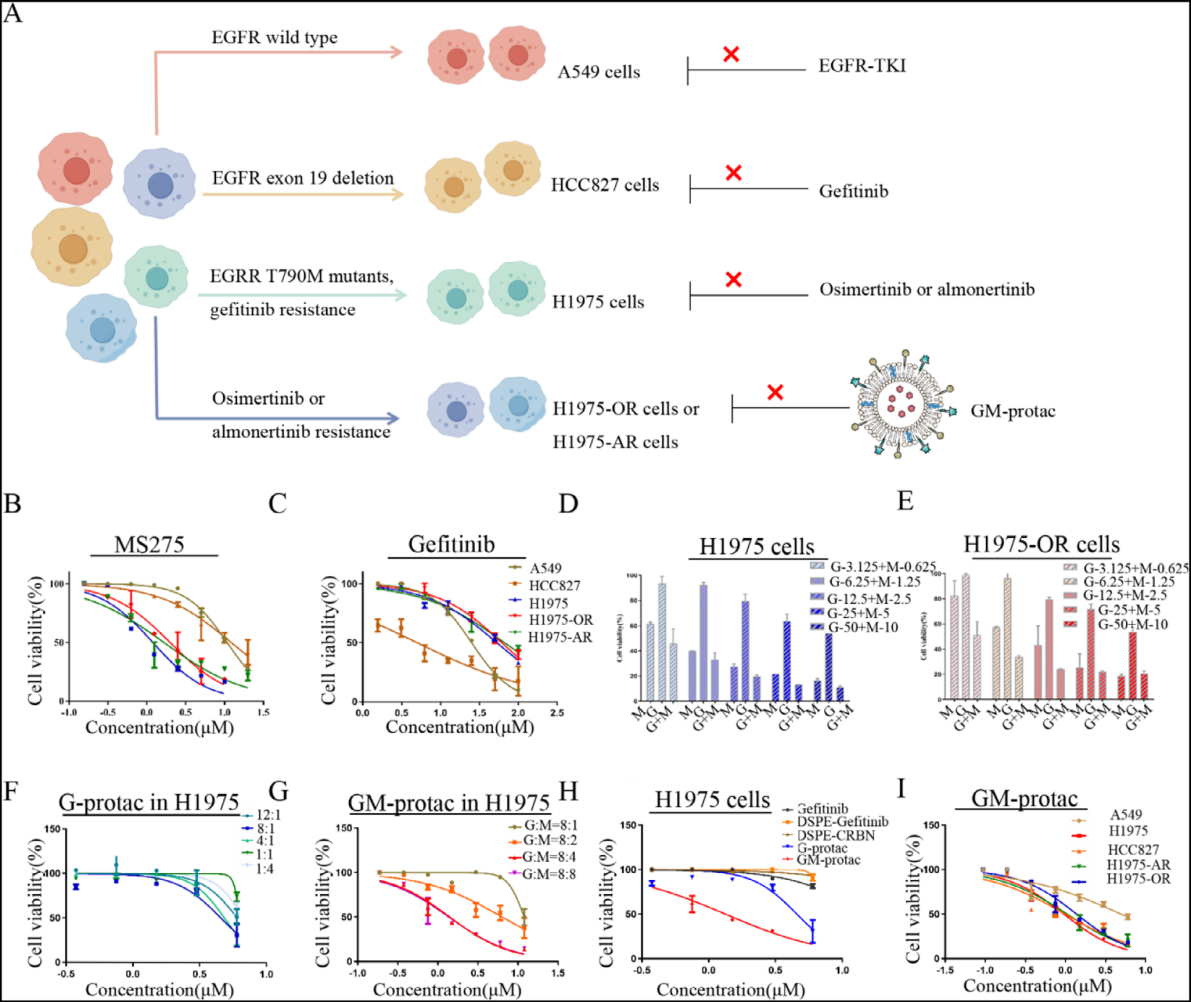
Cell toxicity of different drugs on various lung cancer cells. A) Schematic diagram of different lung cancer cells treated with different drugs. B) Cell toxicity of MS‐275 on different lung cancer cells. C) Cell toxicity of gefitinib on different lung cancer cells. D) The combination effect of MS‐275 and gefitinib on H1975 cells. E) The combination toxic effect of MS‐275 and gefitinib on H1975‐OR cells. F) The cell toxicity of different molar ratios of DSPE‐PEG2000‐gefitinib and DSPE‐PEG2000‐CRBN on H1975 cells. All the concentration of G‐protac and GM‐protac was referred to DSPE‐PEG2000‐gefitinib. G) The cell toxicity of different molar ratios of DSPE‐PEG2000‐gefitinib and MS‐275 on H1975 cells. H) The cell toxicity of different drugs on H1975 cells. I) The cell toxicity of GM‐protac on different lung cancer cells. Error bars represent SEMs of at least three independent measurements.

MS‐275 is a class I HDAC selective inhibitor that can inhibit the cellular function of HDAC1/2/3 to prevent malignant cancer proliferation.^[^ ^53^ ^]^ As studies have proven the efficacy of HDAC3 in osimertinib‐resistant lung cancer cells, we chose this drug to investigate its synergistic effect with EGFR degraders. To study whether HDACi MS‐275 and gefitinib had a synergistic effect on EGFR‐TKI resistant lung cancer cells, we first tested the cell toxicity of MS‐275 or in combination with gefitinib in various lung cancer cells harboring different EGFR mutations shown in **Figure** **2**A–C. As shown in Figure 2B, MS‐275 showed superior efficacy in both H1975 cells (gefitinib resistance),^[^ ^54^ ^]^ H1975‐AR cells (almonertinib resistance) or H1975‐OR cells (osimertinib resistance), but a weaker effect on A549 cells (EGFR wild type) or HCC827 cells (gefitinib sensitivity). The resistance index (RI) of H1975‐OR and H1975‐AR was validated by CCK‐8 assays shown in Figure S10 (Supporting Information) and the RI of H1975‐OR is 5.79 (IC50 in H1975‐OR/IC50 in H1975═4.5/0.777═5.79), RI of H1975‐AR is 6.68 (IC50 in H1975‐AR/IC50 in H1975═4.8/0.719═6.68). While gefitinib showed selective toxicity to HCC827, but a weak effect on other cell lines shown in Figure 2C. We then tested the combination efficacy of MS‐275 and gefitinib in H1975 cells or H1975‐OR cells, as shown in Figure 2D,E, the addition of MS‐275 could significantly increase the sensitivity of gefitinib to H1975 or H1975‐OR cells. To further validate the synergistic effect of MS‐275 and gefitinib, we calculated their combination index (CI) in H1975 cells and H1975‐OR cells, shown as Figure S11 (Supporting Information). As expected, CI of these two cells is below 1, indicating the synergistic effect in cancer cells under the tested concentration.

After the validation of the synergistic effect of MS‐275 and gefitinib, we then tested the toxicity of G‐protac with different molar ratios of DSPE‐PEG2000‐gefitinib and DSPE‐PEG2000‐CRBN, and found the proper toxicity is 8:1 shown in Figure 2F. Based on these results, we then screened the proper ratio of MS‐275 in GM‐protac. When the molar ratio of DSPE‐PEG2000‐gefitinib and MS‐275 increased to 8:4 or 8:8, the cellular toxicity of GM‐protac did not change, indicating the optimized ratio was 8:4 shown in Figure 2G. Next, we compared the toxicity of different compounds in different lung cancer cells. As shown in Figure 2H, GM‐protac exhibited the most potent toxicity among G‐protac, gefitinib, DSPE‐PEG2000‐gefitinib, and DSPE‐PEG2000‐CRBN. GM‐protac also exhibited selective toxicity to gefitinib or osimertinib‐resistant lung cancer cells due to HDAC inhibition by MS‐275, as shown in Figure 2I.

### 2.3 Evaluation of the Degradation Activity and HDAC Inhibition Effect In Vitro

**Figure 3:**
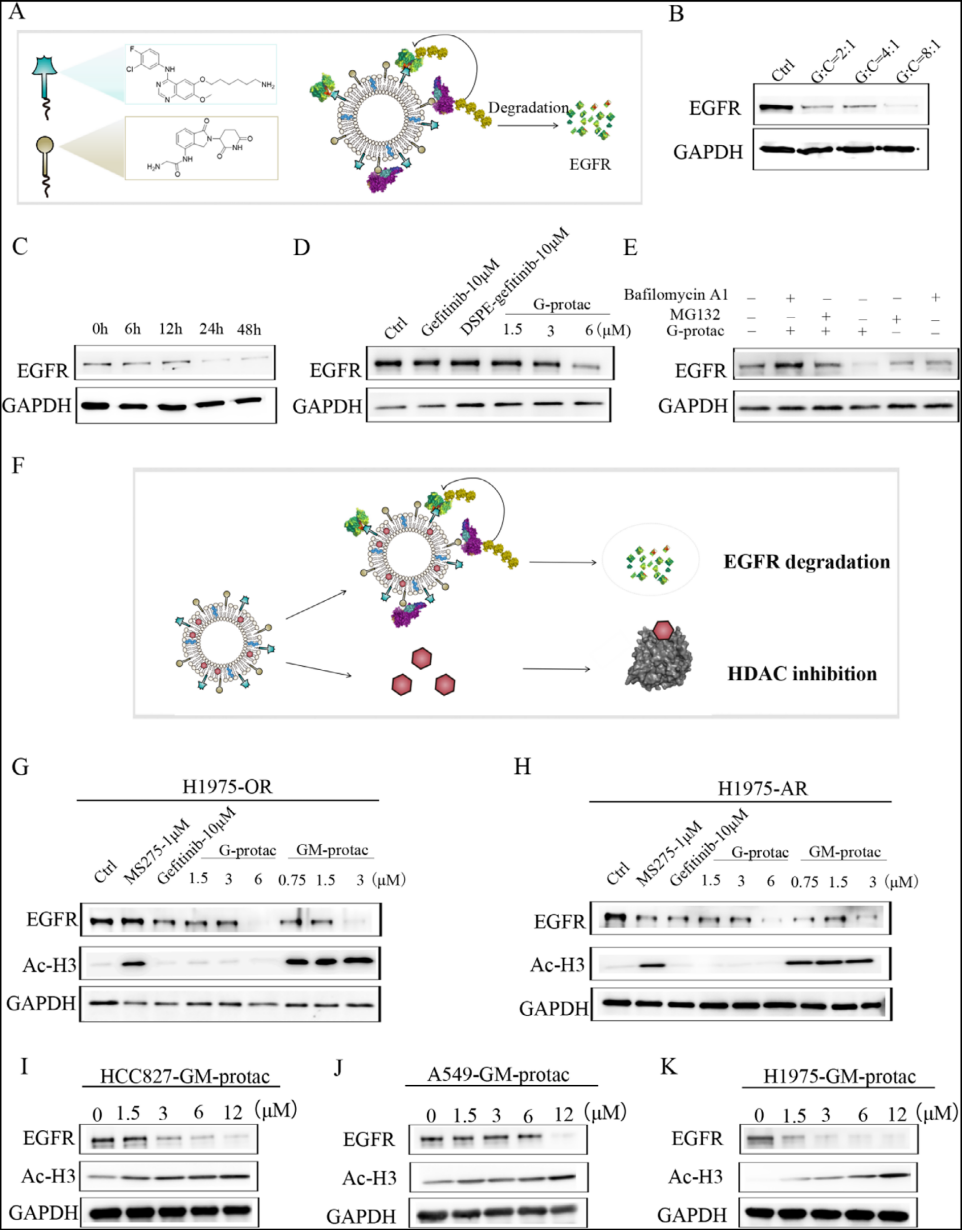
EGFR degradation effect of EGFR lipoSM PROTACs under different conditions. A) Schematic diagram of G‐protac structure and its function on EGFR degradation. B) EGFR degradation of G‐protac (6 µm) with different molar ratios. (C) EGFR degradation of G‐protac (6 µm) at different times. D) EGFR degradation ability by different drugs. E) The mechanisms of EGFR degradation in H1975 cells treated with or without MG132 (0.5 µm) or BalfA1 (0.1 µm). F) Schematic diagram of GM‐protac functions on EGFR degradation and HDAC inhibition. G) EGFR degradation ability of different drugs in H1975‐OR cells. H) EGFR degradation ability of different drugs in H1975‐AR. EGFR degradation effect of GM‐protac on HCC827 cells I) or A549 cells J) or H1975 cells K). All the concentration of G‐protac and GM‐protac was referred to DSPE‐PEG2000‐gefitinib.

After the validation of the optimized molar ratios in GM‐protac, we then evaluate the EGFR degradation activity and HDAC inhibition effect on different lung cancers. First, we tested EGFR degradation by G‐protac on H1975 cells, illustrated in **Figure** **3**A. The EGFR degradation effect of G‐protac with different molar ratios of DSPE‐PEG2000‐gefitinib: DSPE‐PEG2000‐CRBN was tested, and found a ratio of 8:1(DSPE‐PEG2000‐gefitinib: DSPE‐PEG2000‐CRBN) had the best degradation effect on H1975 shown in Figure 3B. Thus, this molar ratio was chosen for the preparation of G‐protac in the following assays. G‐protac was then found to degrade EGFR mutants in H1975 in a dose or time‐dependent manner shown in Figure 3C,D. To study the degradation mechanism, H1975 cells were treated with G‐protac in the presence or absence of proteasome inhibitor MG132. EGFR belongs to the cell membrane protein; we then tested whether EGFR can be degraded via lysosome‐autophagy pathway. Thus, H1975 cells were also treated with G‐protac in the presence or absence of autophagy inhibitor bafilomycin A(1) (BafA1). As shown in Figure 3E, the EGFR degradation could be blocked by MG132 and BafA1, suggesting EGFR degradation could be triggered via ubiquitin−proteasome pathway and lysosome‐autophage pathway. Meanwhile, we also observed the colocalization of lysosome and G‐protac by confocal imaging shown in Figure S12 (Supporting Information), further confirming these results.

We then studied the influence of GM‐protac on EGFR degradation and HDAC inhibition in different lung cancer cells illustrated in Figure 3F. GM‐protac could induce EGFR degradation under a lower concentration compared to G‐protac, and inhibit HDAC enzyme activity by increasing the acetylation of HDAC substrate histone 3 in osimertinib or almonertinib resistance lung cancer cells H1975‐OR or H1975‐AR cells. We also found EGFR degradation by GM‐protac was in a time‐dependent manner and lasted for 72 h shown in Figure S13 (Supporting Information). Meanwhile, GM‐protac showed superior degradation effect on both EGFR mutant H1975 cells and HCC827 cells, but weaker effect on wild type A549 cells, indicating the selectivity activity of GM‐protac, shown in Figure 3I–K.

### 2.4 Effects of GM‐Protac on Cellular Function of H1975‐OR Cells

**Figure 4:**
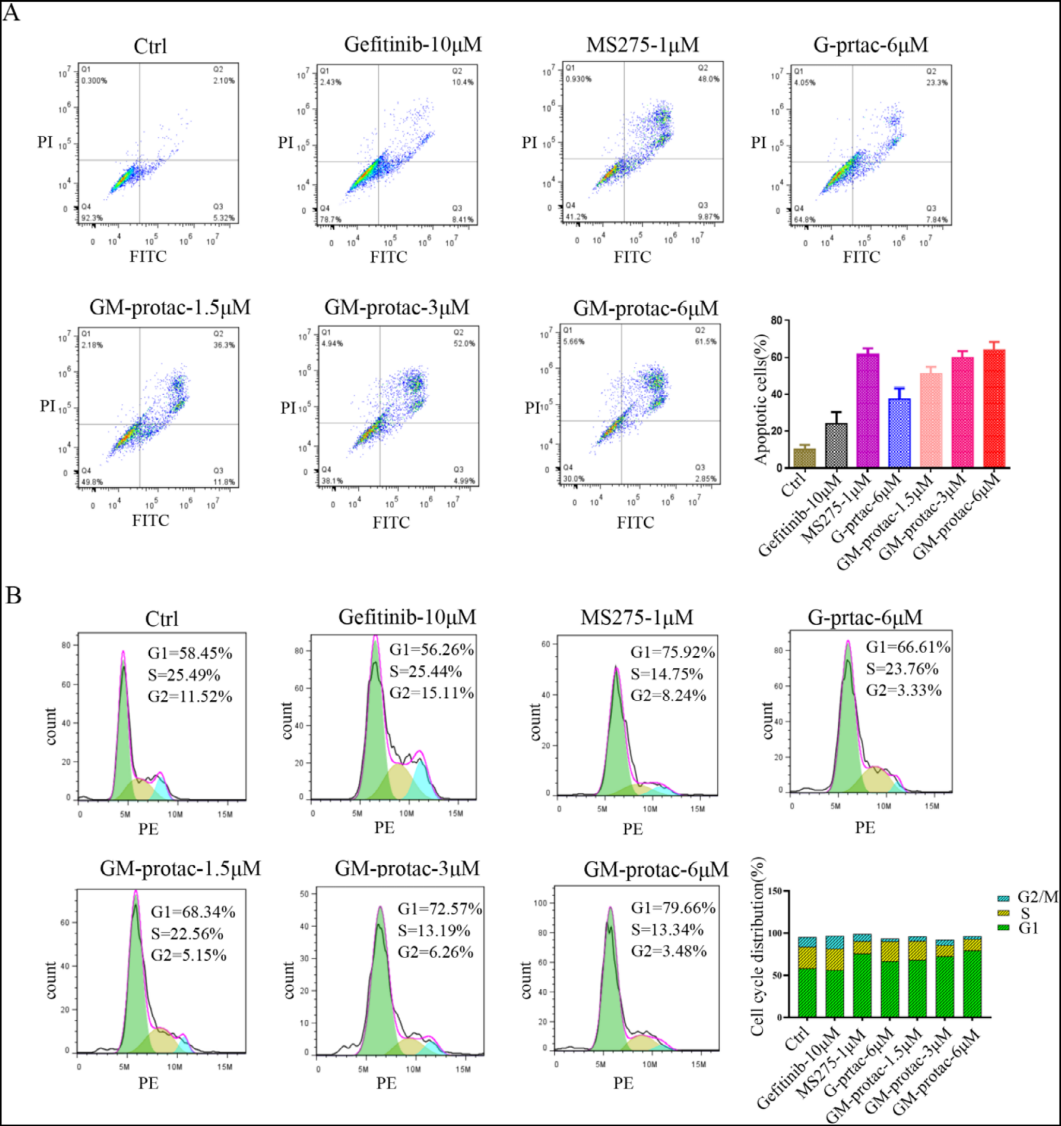
Cellular function influenced by different drugs. A) H1975‐OR cell apoptosis induced by MS‐275 (1 µm), gefitinib (10 µm), G‐protac (6 µm), GM‐protac (1.5, 3, 6 µm). B) Cell cycle arrest of H1975‐OR was modulated by these drugs at different concentrations. Error bars represent SEMs of at least three independent measurements.

HDAC inhibition or EGFR inhibition is reported to be associated with cellular functions such as cell apoptosis and cell‐cycle arrest.^[^ ^23^, ^55^ ^]^ To verify if our developed GM‐protac could also induce cancer cell apoptosis and cell cycle arrest, we treated H1975‐OR cells with different concentrations of GM‐protac and tested by FACS using FITC‐labeled Annexin V and propidium iodide (PI). As shown in **Figure** **4**A, GM‐protac showed an obvious cell apoptosis effect similar to the positive control MS‐275. Compared to G‐protac, GM‐protac had a superior effect even at lower concentrations, indicating the combination efficiency of HDAC inhibition and EGFR degradation. Then we studied their effect on cell cycle arrest using FACS assays. Both MS‐275, and GM‐protac could induce cell cycle arrest in phase G1 more potently than G‐protac shown in Figure 4B. While gefitinib had a weak effect on cell apoptosis and cell cycle arrest due to drug resistance. To sum up, the combination of HDAC inhibition and EGFR degradation by GM‐protac could both induce cell apoptosis and cell cycle arrest at phase G1 in osimertinib resistant lung cancer cells.

### 2.5 Potential Mechanisms of GM‐Protac in Osimertinib Resistance Lung Cancer In Vitro

**Figure 5:**
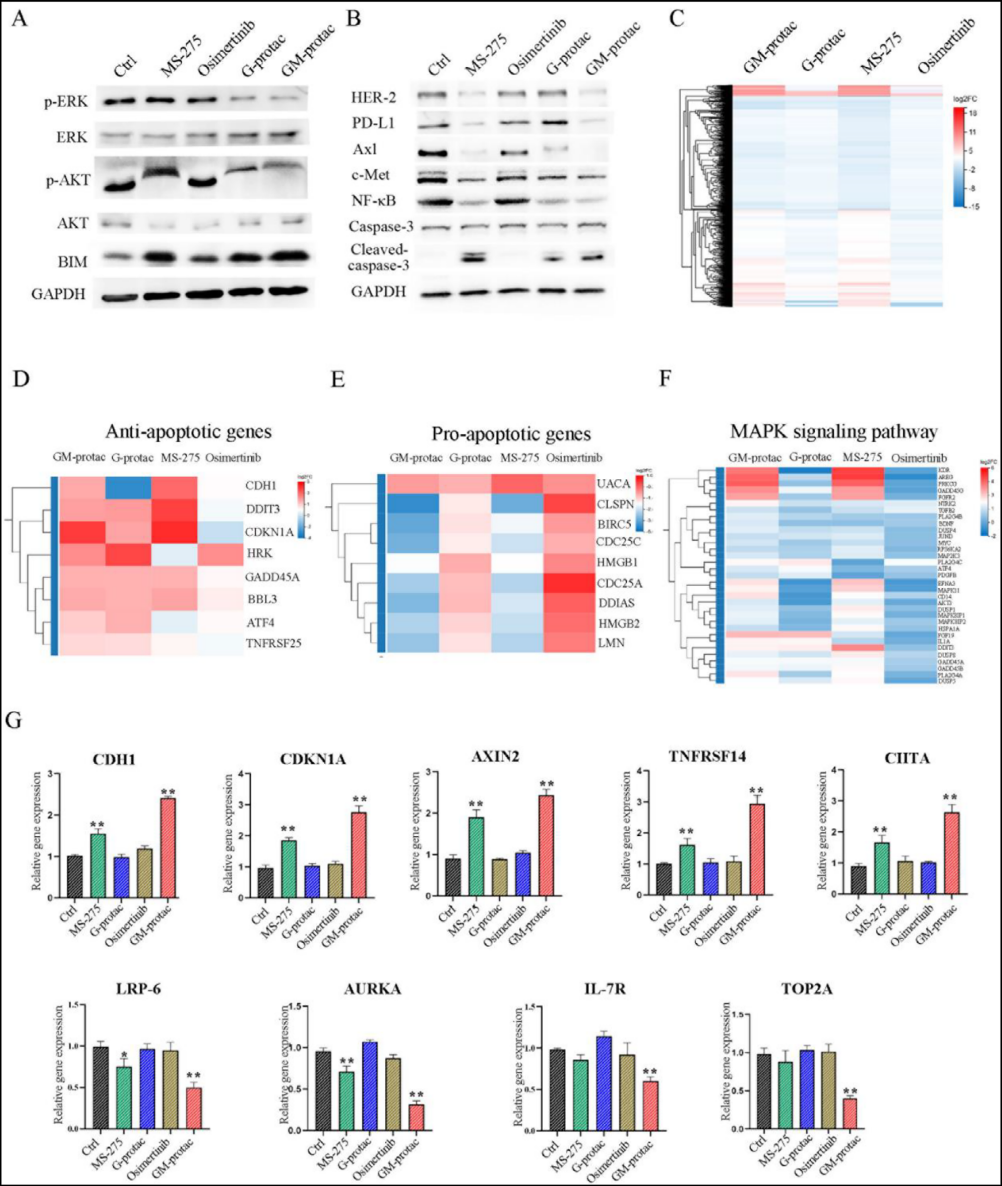
The cellular mechanism of GM‐protac in H1975‐OR cells. A) Western blot analysis of different protein expression on H1975‐OR cells treated with MS‐275 (1 µM), osimertinib (5 µm), G‐protac (6 µm), GM‐protac (3 µm). B) Different protein levels associated with HDAC or EGFR signaling pathway assessed by WB. C) Heat map of differentially expressed genes in RNA‐microarray analysis treated with MS‐275 (1 µm), osimertinib (5 µm), G‐protac (6 µm), GM‐protac (3 µm) in H1975‐OR cells. D)The upregulation of genes associated with pro‐apoptotic signal pathways treated with different drugs. E) The downregulation of genes associated with anti‐apoptotic signal pathways treated with different drugs. F) KEGG enrichment revealed MAPK signaling pathways influenced by different drugs. G) The mRNA levels of cancer‐related genes in H1975‐OR cells incubated with treated with MS‐275 (1 µm), osimertinib (5 µm), G‐protac (6 µm), GM‐protac (3 µm) for 48 h. Fold changes of mRNA levels were analyzed by quantitative PCR. Error bars represent SEMs of at least three independent measurements. ^*^, *P* < 0.05; ^**^, *P* < 0.01 versus control.

After the validation of GM‐protac efficacy on the cellular function of osimertinib‐resistant lung cancer, we then further analyzed the potential mechanisms of GM‐protac on HDAC and EGFR signaling pathways in H1975‐OR cells. As previously reported, HDAC inhibitors could increase the sensitivity of gefitinib and induce cancer cell apoptosis by up‐regulating the level of BIM and activation of caspase‐3,^[^ ^44^ ^]^ we also found this phenomenon by MS‐275 or GM‐protac treatment in H1975‐OR cells shown in **Figure** **5**A,B. Meanwhile, MS‐275 could inhibit PD‐L1 expression, which was also observed in GM‐protac‐treated cancer cells, indicating the HDAC inhibition effect. We also studied the influence of GM‐protac on the EGFR down signal pathway in H1975‐OR. As shown in Figure 5A and G‐protac and GM‐protac could inhibit the activation and phosphorylation of AKT and ERK. Besides, both MS‐275 and GM‐protac could inhibit the expression of HER‐2, Axl, c‐Met, and NF‐κB, but G‐protac had a weaker effect on these proteins, indicating the necessary of HDAC and EGFR signaling pathway inhibition on osimertinib‐resistant lung cancer. The mRNA levels of these proteins were also tested to further support the WB results shown in Figure S14 (Supporting Information).

To explore the effect of GM‐protac on other signal pathways in H1975‐OR cells, we utilized an RNA transcriptome approach for the in‐depth analysis of the associated gene expression. GM‐protac exhibited more gene expression than the G‐protac‐treated or osimertinib‐treated group shown in Figure 5C and Figure S15 (Supporting Information).The gene ontology analysis revealed that the affected genes are associated with many important signaling pathways relative to widely concerning diseases, such as metabolic diseases, cancer, immune diseases, and immune system etc., shown in Figure S16 (Supporting Information). Kyoto Encyclopedia of Genes and Genomes (KEGG) functional enrichment analysis revealed that many important signaling pathways were affected by GM‐protac, such as MAPK signal pathway, the P53 signal pathway, PI3K‐AKT signal pathway shown in Figure S17 (Supporting Information). While other groups had less influence on these signal pathways compared to the GM‐protac group as shown in Figure S17 (Supporting Information).

The deep analysis revealed that GM‐protac could influence the apoptotic signal pathway by up‐regulating the expression of pro‐apoptotic genes and down‐regulating the expression of anti‐apoptotic genes shown in Figure 5D,E. To identify the roles of HDACs in the apoptotic signal pathway, we then analyzed the MS‐275‐treated group and found some overlapped genes with GM‐protac, such as pro‐apoptotic genes DDIT3, CDH1, BBC3, GADD45A, ATF4, TNFRSF25, and anti‐apoptotic genes UACA, HMGB1, HMGB2 etc. While the G‐protac or osimertinib had less effect on these apoptotic signal pathways. Further analysis of the MAPK signaling pathways indicated that both GM‐protac and MS‐275 could influence more associated gene expressions compared to osimertinib group, including the up‐regulation of ATF3, DUSP4, RASGRP3, FGF19 etc., and the down‐regulation of FOXM1, CDK1, and PAK6 shown in Figure 5F. All these data also indicated that GM‐protac could achieve the regulation of the MAPK signal pathway by the combination of EGFR degradation and HDAC inhibition. We also found that GM‐protac and MS‐275 treated groups influenced more gene expression on PI3K‐AKT signaling pathway, RAS signaling pathway, cell cycle signaling pathway, and P53 signaling pathway shown in Figure S18 (Supporting Information).

We then performed RT‐PCR assays to further confirm these results. As shown in Figure 5G, nine related genes were tested for associated with apoptotic or other tumor‐associated pathways. As expected, GM‐protac could significantly increase the expression of tumor suppressor genes such as CDH1, CDKN1A, AXIN2, TNFRSF14, CITTA decrease the expression of oncogenic genes. While other group such as G‐protac or MS‐275 treated group had a much weaker effect. The potential mechanisms suggested that GM‐protac could overcome the EGFR‐TKI resistance by the combination of EGFR degradation and epigenetic regulation of HDACs inhibition.

### 2.6 Evaluation of the Antitumor Activity in TKI‐Resistant Lung Cancer In Vivo

**Figure 6:**
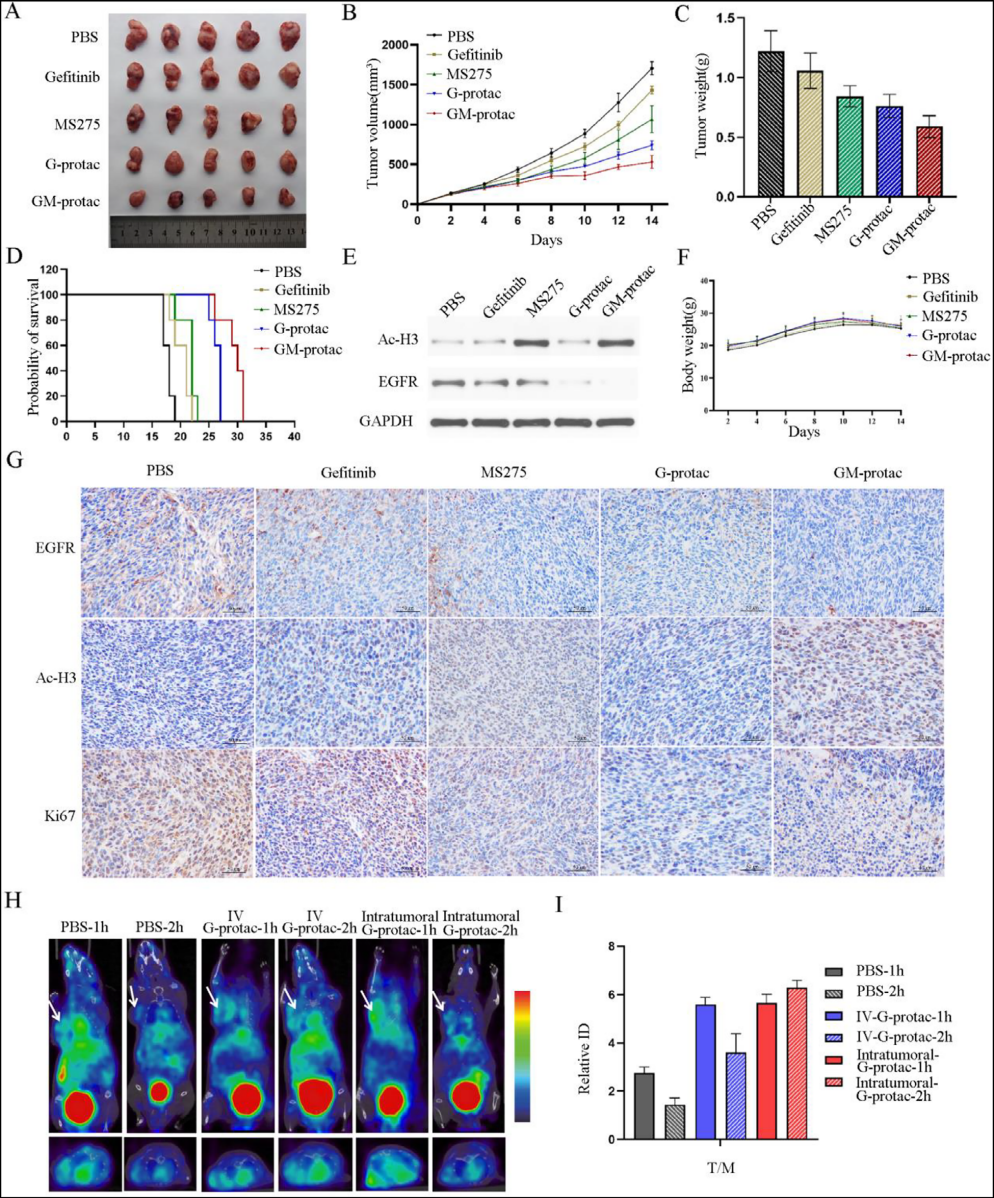
Anti‐cancer effect of different drugs in H1975 lung cancer animal models. A) Tumor tissue image of mice treated with gefitinib (15 mg kg^−1^), MS‐275 (15 mg kg^−1^), G‐protac (15 mg kg^−1^), GM‐protac (15 mg kg^−1^) by tail vein injection. B) Relative changes of tumor volume over time treated by different drugs. C)Tumor weight of mice treated with these drugs. D) Survival curve of lung tumor‐bearing mice treated with these drugs. E) Western analysis of protein expression levels in tumor tissues treated with different drugs. F) Average body growth curves of mice throughout treatment. G) Immunohistochemistry‐stained sections of the tumor tissues after treatment. Data are presented as mean ± SEM (*n* ≥ 5). H) H1975 tumor‐bearing mice were injected with N_3_‐G‐protac 4 h before the injection of ^68^Ga‐DOTA‐DBCO via tail vein injection or intratumoral injection, then Micro‐PET static imaging was performed at 1 and 4 h. I) Biodistribution of ^68^G in tumors in H1975 tumor‐bearing mice at different times after the injection. Data points represent mean ± SD. (*n* = 3).

To investigate the tumor inhibition effect of GM‐protac, we established a subcutaneous xenograft H1975 lung tumor‐bearing mice model. Tumor‐bearing mice were treated with PBS, gefitinib (15 mg kg^−1^), MS‐275 (15 mg kg^−1^), G‐protac (15 mg kg^−1^, the dose of DSPE‐PEG2000‐gefitinib) and GM‐protac (15 mg kg^−1^, the dose of DSPE‐PEG2000‐gefitinib), respectively (10 mice/group, every other day, by tail vein injection) for 3 weeks. As shown in **Figure** **6**A–C, GM‐protac demonstrated the most potent inhibition of tumor at a rate of over 50%, while gefitinib showed the weakest anti‐tumor effect, while MS‐275 showed better inhibition effect than gefitinib, but weaker than G‐protac, indicating the good in vivo behavior of GM‐protac. To further analyze the anti‐tumor effect of these drugs, we then studied whether GM‐protac could prolong the survival of gefitinib‐resistant lung cancer mice. As shown in Figure 6D, GM‐protac could significantly increase the survival of mice with lung cancer compared to the PBS group or other drug‐treated group. G‐protac‐treated mice also had longer survival time than MS‐275 or gefitinib‐treated groups. All these data suggested GM‐protac or G‐protac had a potent *s* anti‐cancer effect.

To analyze their in vivo anti‐cancer mechanisms, we performed western blot assays to evaluate the EGFR degradation effect and HDAC inhibition on lung tumor tissues. As shown in Figure 6E, GM‐protac could induce EGFR degradation and increase the acetylation level of H3 by HDAC inhibition in lung tumor tissues, validating their potent anti‐tumor effect. Drug safety is the most concerning question for drug discovery. We monitored the body weight of mice in each group, and found the body weight didn't change significantly during the drug treatment, indicating the well‐tolerance of GM‐protac in mice shown in Figure 6F. Moreover, the tissues of major organs (heart, liver, spleen, lung, kidney, and brain) were used to evaluate the general toxicity of these formulations using hematoxylin and eosin (H&E) staining. As shown in Figure S19 (Supporting Information), none of the experimental groups displayed obvious organ damages. The in vivo anti‐cancer mechanisms were further analyzed using immuno‐fluorescence experiments which the fluorescence intensity of EGFR or Ac‐H3 in tumor tissues was consistent with the results shown in Figure 6G.

We then tested whether G‐protac had tumor accumulation ability using PET imaging despite the lack of tumor tumor‐targeting ligand. For PET imaging, we used pre‐targeting technology and click chemistry as previously reported.^[^ ^56^ ^]^ DSPE‐PEG2000‐N_3_ was inserted into G‐protac to obtain N_3_‐G‐protac, we chose to inject N_3_‐G‐protac 4 h before the injection of ^68^Ga‐DOTA‐DBCO as we didn't observe obvious tumor accumulation when inject N_3_‐G‐protac 12 h or 24 h before the injection of ^68^Ga‐DOTA‐DBCO as the literature reported.^[^ ^56^ ^]^ We also intratumorally injected N_3_‐G‐protac before the injection of ^68^Ga‐DOTA‐DBCO to make sure they can react in vivo. Micro‐PET static imaging was performed at 1 and 4 h after injection of ^68^Ga‐DOTA‐DBCO. Images indicated that the N_3_‐G‐protac showed obvious uptake on the tumor site at various times compared with PBS, both via tail vein injection or intratumoral injection, as shown in Figure 6H. Also, the liver uptake was very high in consistant with liposome drug distribution. To quantitatively analyze the tumor accumulation of G‐protac, we calculated the tumor‐to‐muscle ratios (T/M) as shown in Figure 6I, the two groups injected with N_3_‐G‐protac showed obvious tumor accumulation compared with the PBS group, indicating the liposomal PROTAC had some tumor accumulation despite the lack of tumor targeting ligand. All these data demonstrated that GM‐protac had a potent in vivo anti‐cancer effect with good biosafety.

**Figure 7:**
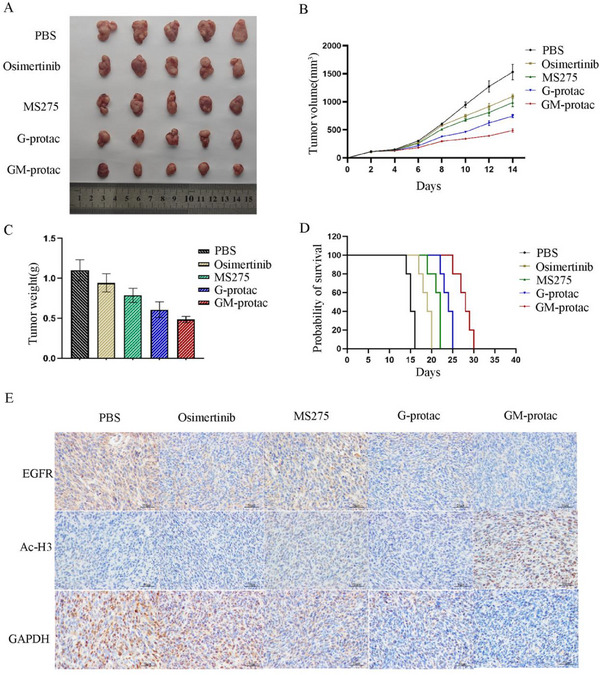
Anti‐cancer effect of different drugs in H1975‐OR lung cancer animal models. A)Tumor tissue image of mice treated with osimertinib (15 mg kg^−1^), MS‐275 (15 mg kg^−1^), G‐protac (15 mg kg^−1^), GM‐protac (15 mg kg^−1^) by tail vein injection. B) Relative changes of tumor volume over time treated by different drugs. C) Tumor weights of mice treated with these drugs. D) Survival curve of lung tumor‐bearing mice treated with these drugs. E) Immunohistochemistry‐stained sections of the tumor tissues after treatment. Data are presented as mean ± SEM (*n* ≥ 5).

The drug resistance of the third‐generation EGFR‐TKI osimertinib is a global problem in lung cancer therapy, we then studied the therapeutic potential of GM‐protac in osimertinib‐resistant lung cancer mice. We established a subcutaneous xenograft H1975‐OR lung tumor‐bearing mice model. Tumor‐bearing mice were treated with PBS, Osimertinib (15 mg kg^−1^), MS‐275 (15 mg kg^−1^), G‐protac (15 mg/kg), and GM‐protac (15 mg/kg), respectively (10 mice/group, every other day, by tail vein injection) for 3 weeks. We treated the mice as the same as the H1975 lung cancer mice described above. As shown in **Figure** **7**A–D, GM‐protac showed a potent anti‐cancer effect and longer survival time with the inhibition rate over 60% compared with other groups. The immuno‐fluorescence experiments revealed that GM‐protac could degrade EGFR and inhibit HDAC activity, similar in H1975 lung cancer mice. Meanwhile, H&E staining and body weight also indicated their in vivo safety shown in Figure S20 (Supporting Information).

All these data demonstrated that our developed degradation system could degrade EGFR mutants and inhibit HDAC activity in both gefitinib‐resistant and osimertinib‐resistant lung tumor tissues and exhibited potent in vivo anti‐cancer effect with good safety. This study revealed a promising strategy to overcome EGFR‐TKI resistance for clinical lung cancer therapy.

## 3 Discussion and Conclusion

EGFR‐TKI resistance is a global challenge urgent to resolve for patients with NSCLC. The EGFR‐TKI resistant mechanisms are complex, mediated by EGFR‐dependent and bypass signaling pathways. Despite the first‐to fourth‐generation EGFR‐TKIs were developed to solve EGFR mutations, drug resistance has not been fundamentally addressed.^[^ ^21^ ^]^ Recent years, drug combination therapy and multiple target inhibitors are considered as promising strategies for osimertinib‐resistant lung cancer.^[^ ^57^ ^]^ For example, a phase 3, multicenter, randomized study revealed that amivantamab (an EGFR‐MET bispecific antibody ) in combination with lazertinib (brain‐penetrant third‐generation EGFR‐TKI), demonstrated antitumor activity in osimertinib‐relapsed cancer patients.^[^ ^58^ ^]^ Another clinical trial revealed that MET‐inhibitor and EGFR‐TKI combination therapy could overcome acquired MET‐mediated osimertinib resistance in NSCLC patients.^[^ ^59^ ^]^ In addition, many other drug combination researches have been conducted to evaluate the safety and efficacy in EGFR‐TKI‐resistant lung cancer patients, such as osimertinib combined with selumetinib (MEK1/2 inhibitor), savolitinib (MET inhibitor), or durvalumab (anti‐PD‐L1 monoclonal antibody).^[^ ^60^ ^]^ Besides drug combination therapy, PROTACs showed great promise to overcome EGFR‐TKI resistance by selectively and efficiently degrading EGFR mutants in lung cancer cells. Nowadays, numerous EGFR targeting PROTACs have been developed based on the extensive screening of EGFR ligands, linkers or E3 ligands, and some of which could efficiently degrade EGFR mutants in gefitinib‐resistant or osimertinib‐resistant lung cancer cells.^[^ ^21^ ^]^ However, these small PROTACs are usually limited to poor pharmaceutical properties, such as poor cell penetration and tumor targeting ability.

To address these challenges, we designed an EGFR lipoSM PROTAC platform for efficient EGFR degradation, simply constructed by liposome self‐assembly. Besides, the degradation efficiency of EGFR lipoSM PROTAC was screened by adjusting the molar ratio of DSPE‐PEG2000‐gefitinib and DSPE‐PEG2000‐CRBN, rather than screening the linker length or the structure of E3 ligand and EGFR ligand, like small PROTACs. To block the EGFR‐dependent signal pathways and bypass resistance mechanisms, we use this platform as a vehicle to encapsulate MS‐275 (GM‐protac) to enhance the sensitivity of the EGFR degrader to osimertinib due to the function of HDACs in EGFR‐TKI‐resistant lung cancer cells.^[^ ^44^ ^]^ As expected, the HDAC inhibitor showed selective toxicity toward H1975 cells or H1975‐OR cells compared with HCC827 cells or A549 cells, indicating the important roles of HDACs in EGFR‐TKI‐resistant lung cancer cells. GM‐protac also showed potent toxicity to EGFR‐TKI‐resistant lung cancer cells, such as H1975 cells, H1975‐OR cells, and H1975‐AR cells. Further mechanism analysis revealed that GM‐protac could both inhibit HDAC and EGFR signaling pathways, such as influencing BIM‐associated apoptosis, cell cycle arrest, and downregulation of HER2, c‐Met, Axl, and the PI3K‐AKT signaling pathways etc. GM‐protac also demonstrated potent in vivo tumor inhibition (50−60% inhibition rate) and negligible toxicity in both gefitinib and osimertinib‐resistant lung cancer mice. These results further validated that our developed PROTAC system had good biocompatibility and promising potential for clinical translation for EGFR‐TKI‐resistant lung cancer therapy. Meanwhile, as liposome is the approved nanoparticle for drug delivery, the “split and mix ” liposomal PROTACs, could be a promising platform for drug combination therapy, taking advantage of the good pharmaceutical properties of liposomes and targeted protein degradation by PROTAC.

## 4 Experimental Section

### Materials

All solvents and reagents used for compound synthesis were purchased from Shanghai Lingfeng Chemical Reagents Co., Ltd (Shanghai, China) and used without further purification. Methanol and acetonitrile were purchased from Thermo Fisher Scientific (Shanghai, China). HSPC (hydrogenated soy phosphatidylcholine), DSPE‐PEG2000‐NHS (1,2‐distearoyl‐sn‐glycero‐3‐phosphoethanolamine N‐[succinimidyl (polyethylene glycol)‐2000]), mPEG2000‐DSPE (N‐ (methoxyl polyethylene glycol 2000)−1,2‐distearoyl‐sn‐glycero‐3‐phosphoethanolamine), and cholesterol were purchased from A.V.T. Pharmaceutical Co., Ltd (Shanghai, China). All the antibodies against EGFR, AKT, phospho‐AKT, ERK, phospho‐AKT, Ac‐H3, GAPDH, BIM, cleaved caspase‐3, caspase‐3, HER‐2, Axl, NF‐κB, c‐Met, and H3 etc. were obtained from Proteintech. Reagents used for biological assays were purchased from Sigma–Aldrich and Thermo Fisher. Cells were purchased through ATCC and cultured according to ATCC guidelines.

### Cell Lines and Cell Culture

Human lung cancer cell lines, A549 (RRID: CVCL_0023), HCC827(RRID:CVCL_2063), H1975 (RRID: CVCL_1511) were obtained from ATCC. The osimertinib‐resistant H1975‐OR cells were established in our laboratory by exposing H1975cells to gradually increasing concentrations of osimertinib (starting at 10 nm and ending with 1000 nm) for ≈3 months.^[^ ^54^ ^]^ The almonertinib‐resistant H1975‐AR cells were also established in our laboratory by exposing H1975 cells to gradually increasing concentrations of almonertinib (starting at 30 nm and ending with 1000 nm) for ≈3 months as previously reported.^[^ ^61^ ^]^ All these cells were cultured in 1640 with 10% (v/v) fetal bovine serum (FBS) and penicillin/streptomycin (100 g mL^−1^). In addition, H1975‐OR cells were cultured in 1640 containing 10% FBS, penicillin/streptomycin (100 g mL^−1^), and osimertinib (1 µm). Similarly, H1975‐AR cells were cultured in 1640 containing 10% FBS, penicillin/streptomycin (100 g mL^−1^), and almonertinib (1 µm). All reagents were purchased from Gibco. All these cells were maintained in a humidified incubator containing 5% CO2 at 37 °C.

### Animals

BALB/c nude were purchased from the Hubei Provincial Center for Disease Prevention and Control, Wuhan, China. All animals were fed and experimented in accordance with the requirements of the Huazhong University of Science and Technology (HUST)'s ethical guidelines for laboratory animals.

### Synthesis of DSPE‐PEG2000‐Gefitinib and DSPE‐PEG2000‐CRBN

DSPE‐PEG2000‐modified drugs were synthesized according to previously reported work,^[^ ^33^ ^]^ and the detailed processes are obtained in Supporting Information.

### Preparation of Liposome‐Based PROTACs

All liposome‐based PROTACs were prepared by the thin film hydration and extrusion method, as previously reported.^[^ ^33^ ^]^ For G‐protac without MS‐275 encapsulation, the mixture of HSPC/Cholesterol/DSPE‐PEG2000‐gefitinib/DSPE‐PEG2000‐CRBN(50/40/8/2, by molar ratio) was dissolved in chloroform and removed by evaporation to obtain the dried lipid film, followed by hydration with ultrapure water at 65 °C for 1 h, and the ratio was referenced by other published work.^[^ ^33^ ^]^ The lipid solution was extruded 7 times through polycarbonate filters (400 and 1000 nm pore size) using an Avanti extruder (Avanti Polar Lipids, USA), then the liposomes were purified by centrifuging third times at 2500 rpm for at least 10 min each using a 100 KD ultrafiltration tube. To optimize the molar ratio for G‐protac preparation, a regression equation was first made of DSPE‐PEG2000‐gefitinib at an absorption wavelength of 330 nm, *y* = 0.0166x + 0.3646, and the regression coefficient standard curve is *R* ^2^ = 0.9998. Then the molar ratio of HSPC/Cholesterol/DSPE‐PEG2000‐gefitinib/DSPE‐PEG2000‐CRBN at 50/40/8/2, 50/40/12/2, 50/40/16/2 was screened, the drug loading efficiency (LE) was analyzed absorption wavelength of 330 nm by ELIASA according to the formulations: LE (%) = (W_T_ – W_S_)/W_T_ * 100%, W_T_ represented the total weight of drugs, and W_S_ represented the weight of drugs in the supernatant. The GM‐protac encapsulated with MS‐275 was prepared with the same procedure. Briefly, a mixture of HSPC/Cholesterol/DSPE‐PEG2000‐gefitinib/DSPE‐PEG2000‐CRBN/MS‐275 with different molar ratios was dissolved in chloroform and removed by evaporation to obtain the dried lipid film, followed by hydration and extrusion, and purification as described above. The drug loading efficiency (LE) of MS‐275 was analyzed as the same procedure of gefitinib at the absorption wavelength of 240 nm by ELIASA.

### Characterization of Liposome‐Based PROTACs

The various liposome‐based PROTACs were characterized by a Zetasizer (Malvern Instruments, UK) and transmission electron microscope (TEM) (Jem‐1400 Plus, Japan).

### Cell Viability Assay

The cytotoxic activity of drugs or liposome‐based PROTACs to different lung cancer cells for 24 h was assessed by the CCK‐8 assay (Cell Counting Kit‐8) (Biosharp). Briefly, cells were incubated on a 96‐well plate for 24 h in growth medium before drug treatment. Then, different concentrations of drugs or liposome formulations (calculated based on gefitinib concentration) in the 10% FBS medium were added and incubated with cells for 24 h. Then 10 µL CCK‐8 regent was added into each well and incubated for another 1 h at 37 °C. The absorbance was measured at 450 nm by ELISA (ENVISION Nexus).

### Western Blotting

For western blot analysis, different cancer cells were seeded in 12‐well plates with 24 h incubation, then treated with different drugs or liposome‐based PROTACs at different concentrations or different times. After incubation, cells were washed with PBS, harvested, and then lysed using RIPA lysis buffer (Biosharp). The protein concentration was determined by bicinchoninic acid (BCA) protein assay kit (Elabscience). An equal amount of protein was loaded onto an SDS‐PAGE gel and resolved by electrophoresis. Protein bands were then transferred to a polyvinylidene fluoride membrane (PVDF) by a wet transfer cell (Bio‐Rad, USA). Then the membranes were blocked in 5% skim milk (dissolved in 1 × TBST buffer: 1.5 m NaCl, 20 mm Tris‐HCl, 0.05% Tween‐20) for 1 h and were incubated with an appropriate primary antibody overnight at 4 °C. Then, the membranes were washed with 1 × TBST buffer. Secondary antibodies of goat anti‐rabbit or anti‐mouse were used for 1 h incubation at room temperature. Proteins were then visualized with chemiluminescent substrates.

### Cell Apoptosis and Cell Cycle Assay

The cell apoptosis assay was performed by flow cytometry analysis. H1975‐OR cells were seeded in each well of the 12‐well plates for 24 h. Then, different drugs were added to the medium with 10% FBS and incubated with cells for 24 h. After incubation, the cells were harvested by trypsinization and washed twice with PBS, and suspended in 1× binding buffer. Then the suspended cells were stained with FITC‐labeled Annexin V and propidium iodide (PI) using the Annexin V‐FITC Apoptosis Detection Kit (Biosharp, BL107A). Finally, apoptotic cells were detected and analyzed by flow cytometry. As for the cell cycle arrest experiments, H1975‐OR cells were seeded in a 12‐well plate and grow for 24 h in medium with 10% FBS. Then different drugs were treated in a medium of 10% FBS and incubated with cells for 12 h. Then, the cells were washed twice with PBS and harvested by trypsinization. The cells were fixed with cold 70% ethanol for 4 h and isolated by centrifugation at 2000 rpm for 10 min. Then, the precipitant cells were suspended in PBS with PI staining and 1 mg mL^−1^ RNase at 37 °C for 30 min using the Cell Cycle Analysis Kit (Elabscience). The samples were detected by a flow cytometer, and the percentages of cells in G0‐G1, S, and G2–M phases were analyzed by FlowJo software.

### RNA Isolation and Quantitative RT‐PCR

H1975‐OR cells were seeded in 12‐well plates with 24 h incubation, then treated with osimertinib (5 µm), MS‐275 (1 µm), G‐protac (6 µm), or GM‐protac (3 µm) for another 48 h. The cells were harvested, and total RNA was extracted from H1975‐OR cells using TRIzol reagent (Invitrogen). The isolated mRNA was quantified by Nano‐Drop (ND‐2000). The mRNA levels of the target genes were detected by real‐time PCR using SYBR green (Takara) in an ABI Prism 7500 real‐time PCR system (Applied Biosystems). The data represent two independent experiments with three technical replicates.

### In Vivo Imaging

When the volumes of the H1975 tumor reached 150–250 mm^3^, the BALB/c nude mice were divided into two groups randomly and received an i.v. injection of 200 µL PBS or N_3_‐labeled G‐protac. PET imaging was performed on a micro‐PET (TransPETBioCaliburnLH, Raycan Technology Co., Ltd., Suzhou, China). As for the preparation of N_3_ labeled G‐protac, a mixture of HSPC/Cholesterol/DSPE‐PEG2000‐gefitinib/DSPE‐PEG2000‐CRBN/DSPE‐PEG2000‐N_3_ with different molar ratios (50/40/8/2/2) was dissolved in chloroform and removed by evaporation to obtain the dried lipid film, followed by hydration and extrusion, and purification as described above. To make sure the N_3_‐G‐protac could be successfully labeled with ^68^Ga‐DOTA‐DBCO in vivo, N_3_‐G‐protac as a positive control was intratumorally injected.^[^ ^56^ ^]^ To test the tumor distribution of liposomal protac, N_3_‐G‐protac was intravenously injected into mice, then 4 h after the injection, ^68^Ga‐DOTA‐DBCO was subsequently injected into the tumor‐bearing mice via the tail vein. PET static imaging was performed at 1s and 2 h after the injection of ^68^Ga‐DOTA‐DBCO.

### In Vivo Antitumor Efficacy in a Mouse Tumor Xenograft Model

Balb/C nude mice (15–16 g, 5–6 weeks old) were obtained from Vital River Laboratory Animal Technology Co. Ltd. (Beijing, China) and allowed an acclimation period of 1 week in an isolated biosafety facility for specific pathogen‐free animals. All operations were carried out in accordance with the requirements of the Huazhong University of Science and Technology (HUST)’s ethical guidelines for laboratory animals. To evaluate in vivo anti‐tumor effect, H1975 or H1975‐OR cells were first harvested and resuspended in 1640 without FBS at a density of 1 × 10^8^ mL^−1^. Then, Balb/C nude mice were inoculated with 1 × 10^7^ H1975 cells or H1975‐OR cells (100 µL/each) subcutaneously in the lower flank of mice. When the tumor volume reached about 100–150 mm^3^, H1975 bearing mice were randomly divided into five groups of 10–11 mice per treatment group and were injected with PBS, gefitinib (15 mg kg^−1^), MS‐275 (15 mg kg^−1^), G‐protac (15 mg kg^−1^), GM‐protac (15 mg kg^−1^) via intravenous injection every two days. As for H1975‐OR bearing tumor animal model, mice were randomly divided into five groups of 10–11 mice per treatment group and were injected with PBS, osimertinib (15 mg kg^−1^), MS‐275 (15 mg kg^−1^), G‐protac (15 mg kg^−1^), GM‐protac (15 mg kg^−1^) via intravenous injection every two days. Tumor volumes and body weight were recorded every two days and calculated using the following formula: V (mm^3^) = L × W^2^ /2 (W being the shortest dimension and L the longest dimension). At 21 days’ treatment, mice were sacrificed, and tumors and major organs (heart, liver, spleen, lung, kidney, and brain) were collected and fixed in 4% paraformaldehyde for histologic experiments. The H&E staining slices were examined under a light microscopy (Olympus BX51). For the IHC assay, the lung tumor tissue microarray slides were immersed in 3% H_2_O_2_ for 5 min to inactivate the endogenous peroxidase. Antibodies against EGFR and Ac‐H3 were used to test protein expression in tumor tissue after the treatment with these different drugs. To further confirm these results, WB assays were carried out to estimate the EGFR degradation effect and HDAC inhibition. The time of death of each mouse was tracked and recorded, and Kaplan‐Meier survival curves were constructed for these two lung cancer bearing mice models.

## Conflict of Interest

The authors declare no conflict of interest.

## Author Contributions

D.W contributed to conceptualization, methodology, formal analysis, investigation, wrote the original draft. Y.L. contributed to methodology, formal analysis, and validation. Y.C. contributed to methodology, validation, and investigation. C.D. contributed to methodology, validation, and investigation. W.Z.H. contributed to methodology, validation, and investigation. J.H. contributed to investigation and methodology. Z.L. acquired resources and performed conceptualization. F.Y. contributed to conceptualization, methodology, data curation, formal analysis, and investigation. Y.Z. acquired resources and contributed to formal analysis, validation, and supervision. C.S. contributed to conceptualization, validation, methodology, data curation, and wrote, reviewed and edited the final manuscript.

## Supporting information



Supporting Information

## Data Availability

The data that support the findings of this study are available from the corresponding author upon reasonable request.
