# Supplementary material for: EGFR Targeted Liposomal PROTAC Assisted With Epigenetic Regulation as an Efficient Strategy for Osimertinib‐Resistant Lung Cancer Therapy
Source: Adv Sci (Weinh). 2025 Aug 21;12(43):e10197. doi: 10.1002/advs.202510197 (PMC12631885; doi:10.1002/advs.202510197)
Supplement: Supplementary file 1 — Supporting Information [file ADVS-12-e10197-s001.docx]

**Supplemental Information**

**EGFR targeted liposomal PROTAC assisted with epigenetic regulation as an efficient strategy for osimertinib-resistant lung cancer therapy**

*Dongyuan Wang^1,2^, Yajing Liu^1,2^, Ying Chen^3,4^, Chuan Dai^3,4^, Wenzhu Hu^5^, Jinyan Han^3,4^, Zigang Li^3,4^, Feng Yin^3,4^, Yu Zhang^1,2^, Chen Shi^1,2^**

Dongyuan Wang, Yajing Liu, Yu Zhang, Chen Shi

Department of Pharmacy, Union Hospital, Tongji Medical College, Huazhong University of Science and Technology, Wuhan 430022, China; Hubei Province Clinical Research Center for Precision Medicine for Critical Illness, Wuhan 430022

Email: shichen@hust.edu.cn

Ying Chen, Chuan Dai, Jinyan Han, Zigang Li, Feng Yin

State Key Laboratory of Chemical Oncogenomics, School of Chemical Biology and Biotechnology, Peking University Shenzhen Graduate School, Shenzhen 518055, China; Pingshan Translational Medicine Center, Shenzhen Bay Laboratory, Shenzhen 518118, China.

Wenzhu Hu

Department of Nuclear Medicine, Union Hospital, Tongji Medical College, Huazhong University of Science and Technology, Wuhan 430022, China.

1. **Methods**

**1.1 The synthesis of Gefitinib derivative**

A mixture of N-Boc-6-Bromohexylamine (840 mg, 3 mmol, 1.5 eq.) and 4-(3-Chloro-4-fluorophenylamino)-7-methoxyquinazolin-6-ol (640 mg, 2mmol, 1.0 eq.), together with K2CO3 (828 mg, 6 mmol, 3.0 eq.) in DMF（6 mL）were stirred at ambient temperature for 14h. Then water (30 mL) was added and the mixture was extracted with ethyl acetate (30 mL × 3) and washed with brine (40 mL), dried over anhydrous Na_2_SO_4_, filtered, and concentrated in vacuo. The residue was purified by silica gel (petroleum ether/EtOAc = 4/1) to give S1 as a yellow solid (849 mg, 82% yield). Then S1 (849 mg, 1.64 mmol) was dissolved in DCM (20 mL) which included 10% TFA, stirred at room temperature for 2 h. Then the solution was concentrated in vacuo without further purification to obtain 1 (846 mg, 97% yield) as a yellow powder. ESI-MS: ([M + H]+): 419.1642. MP: 178-180℃.

^1H^ NMR (500 MHz, DMSO-d6) δ 10.85 (s, 1H), 8.79 (s, 1H), 8.00 (d, J = 2.5 Hz, 1H), 7.99 (d, J = 1.6 Hz, 1H), 7.78 (s, 3H), 7.70 (ddd, J = 9.0, 4.3, 2.6 Hz, 1H), 7.53 (t, J = 9.1 Hz, 1H), 7.33 (s, 1H), 4.15 (t, J = 6.5 Hz, 2H), 3.99 (s, 3H), 2.84 – 2.74 (m, 2H), 1.89 – 1.77 (m, 2H), 1.64 – 1.51 (m, 2H), 1.50 – 1.44 (m, 2H), 1.44 – 1.34 (m, 2H).

^13C^ NMR (101 MHz, DMSO-d6) δ 158.6, 157.8, 156.2, 153.6, 149.8, 149.4, 134.8, 126.0, 124.7, 119.4, 118.5, 117.1, 115.5, 107.6, 103.4, 101.7, 69.0, 56.5, 38.8, 28.3, 27.0, 25.6, 25.1.

**1.2 The Synthesis of DSPE-PEG2000-Gefitinib and DSPE-PEG2000-CRBN**

DSPE-PEG2000-modified drugs were synthesized according to previously reported work^[1]^. In detail, the DSPE-PEG2000-NHS and gefitinib derivates or CRBN ligand derivates were dissolved in dichloromethane (DCM) at the molar ratio of 1:1 and stirred with a 2-equivalent concentration of DIPEA (N, N-Diisopropylethylamine) at room temperature for 24 h. Then, the mixture was evaporated and added with 1-5 mL N, N-Dimethylformamide (DMF) for later use. Next, the mixture was purified by membrane dialysis in a dialysis bag with a molecular weight cutoff of 1000 Da. Thus, the solvent DMF was replaced with ultrapure water at room temperature for 72 h. After freeze-drying, a white powder product was stored at −20 ℃ until use.

1. **Supplementary Figures**

**
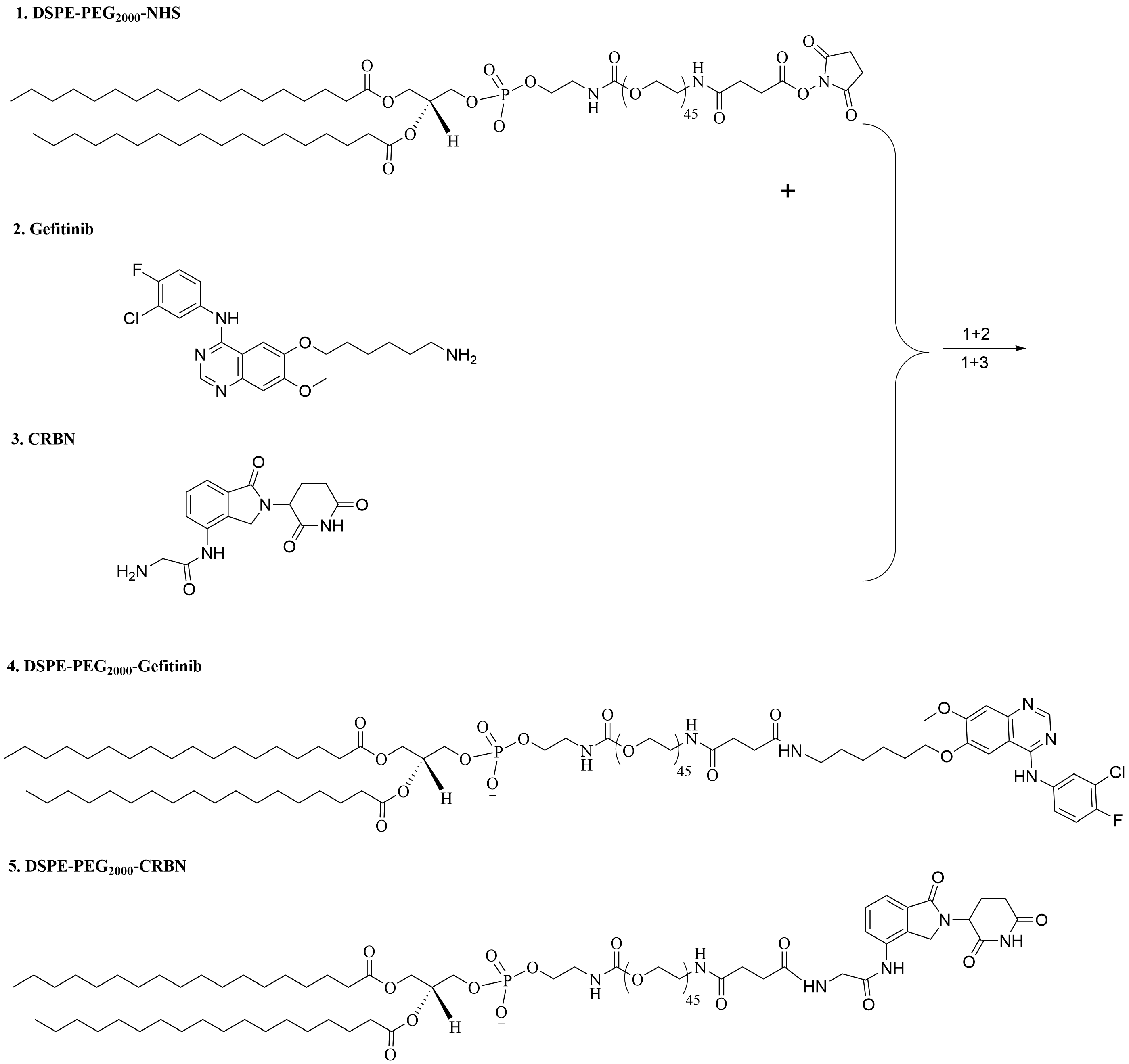
**

**Figure S1.** Synthesis route of DSPE-PEG2000-gefitinib and DEPE-PEG2000-CRBN**.**

**Figure S2.** ^1^H NMR of gefitinib derivates

**Figure S3.** ^13^C NMR of gefitinib derivates

**Figure S4.** ESI-MS of gefitinib derivates

**Figure S5.** High-resolution mass spectrometry (HRMS) of DSPE-PEG2000-NHS.

**Figure S6.** High-resolution mass spectrometry (HRMS) of DSPE-PEG2000-CRBN.

**Figure S7.** High-resolution mass spectrometry (HRMS) of DSPE-PEG2000-gefitinib.


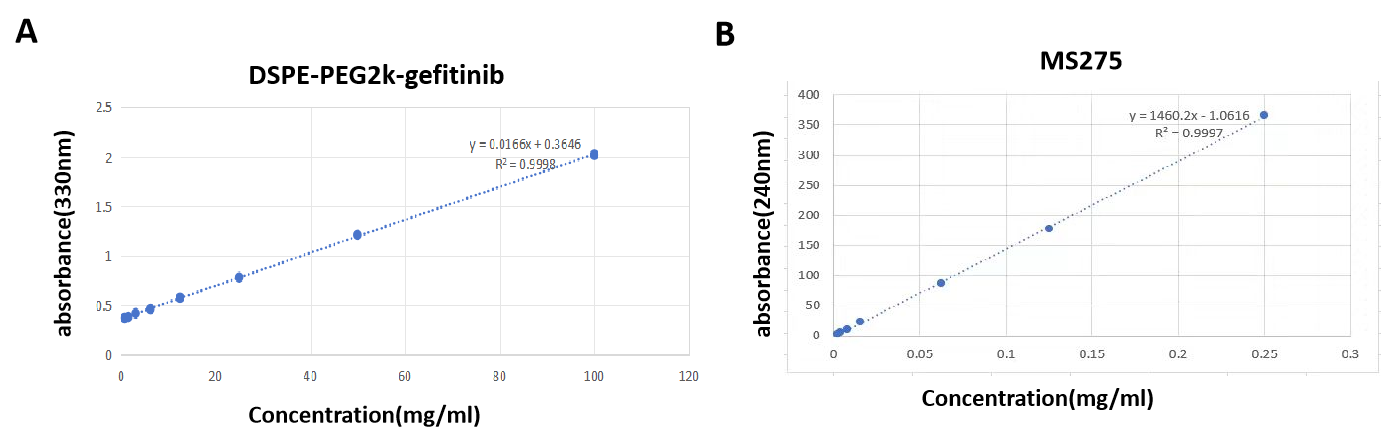


**Figure S8.** Standard curve of DSPE-PEG2000-gefitinib(A) and MS275(C), and the encapsulation efficiency(%) of DSPE-PEG2000-gefitinib(B) and MS275(D) in GM-protac liposomes.


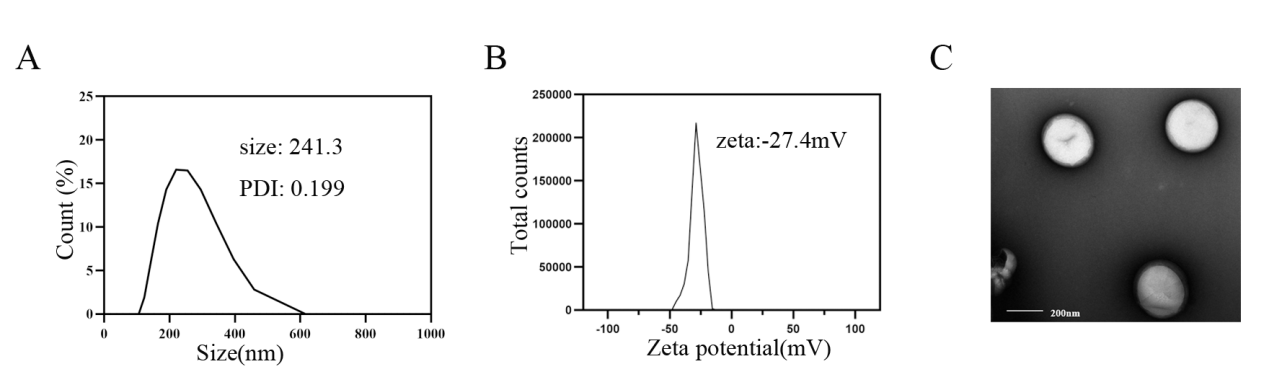


**Figure S9.**  The characterization of GM-protac. (A) Particle size distribution of GM-protac. (C) Zeta potential distribution of GM-protac. (D) TEM image of GM-protac.

**
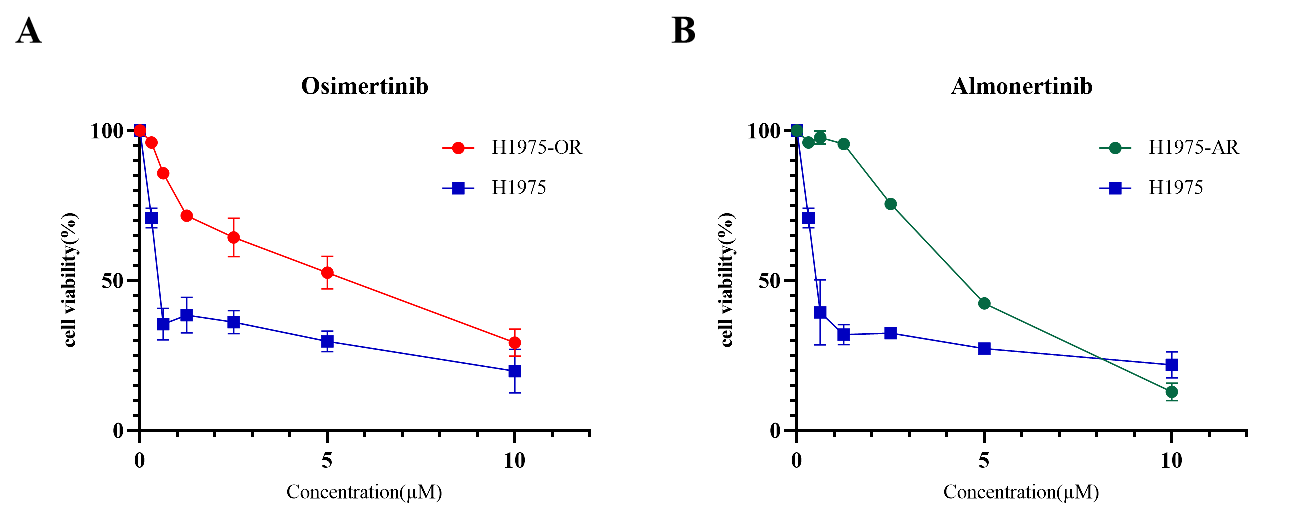
**

**Figure S10.** (A)The cell toxicity of osimertinib in H1975 cells or H1975-OR cells. (B)The cell toxicity of almertinib in H1975 cells or H1975-AR cells.


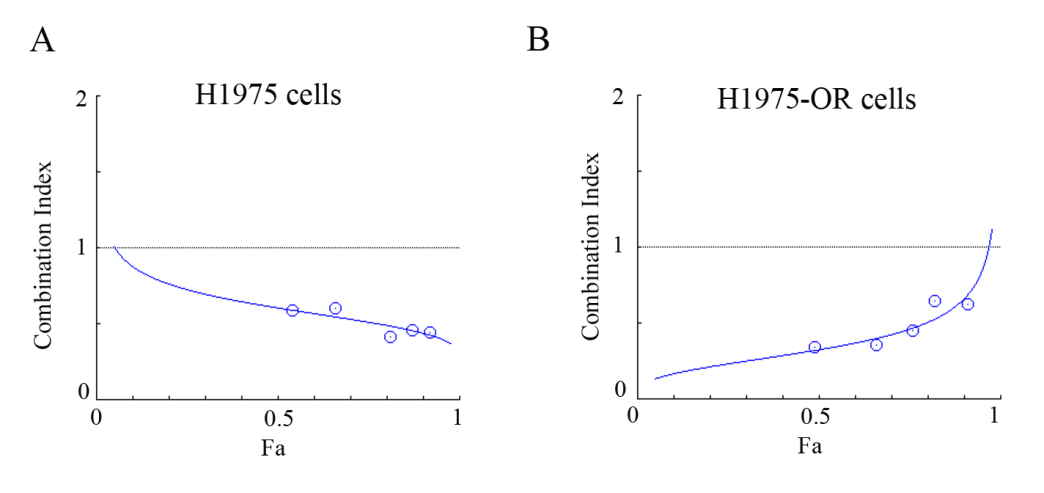


**Figure S11**. The CI index of MS275 and gefitinib combination in H1975 cells (A) and H1975-OR cells (B).


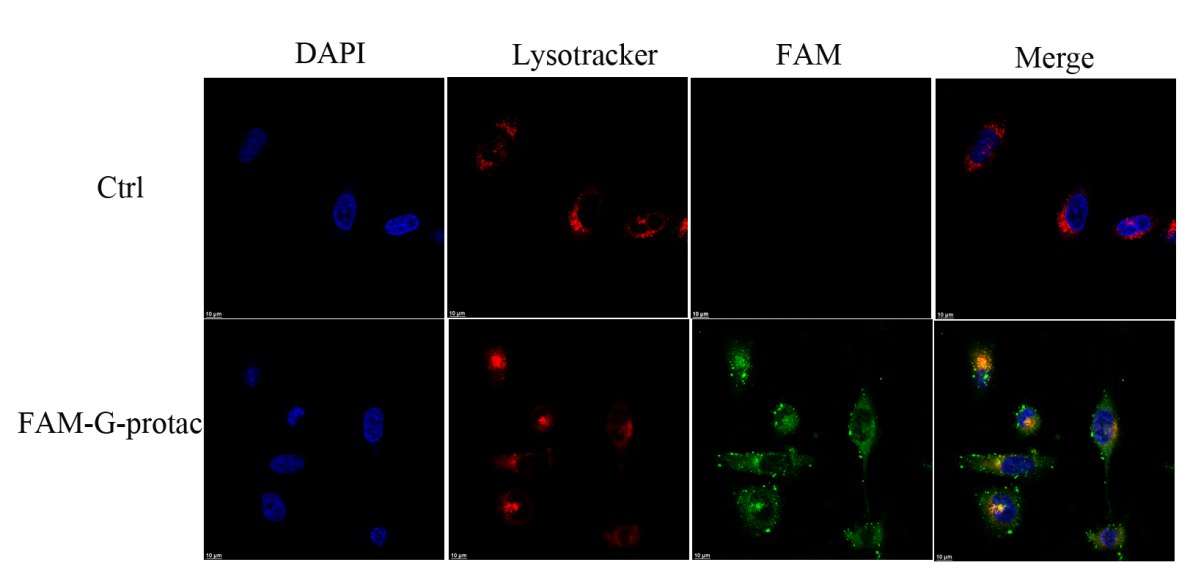


**Figure S12**. The colocalization of lysosome and G-protac labelled with DSPE-PEG2000-FAM by confocal imagine.


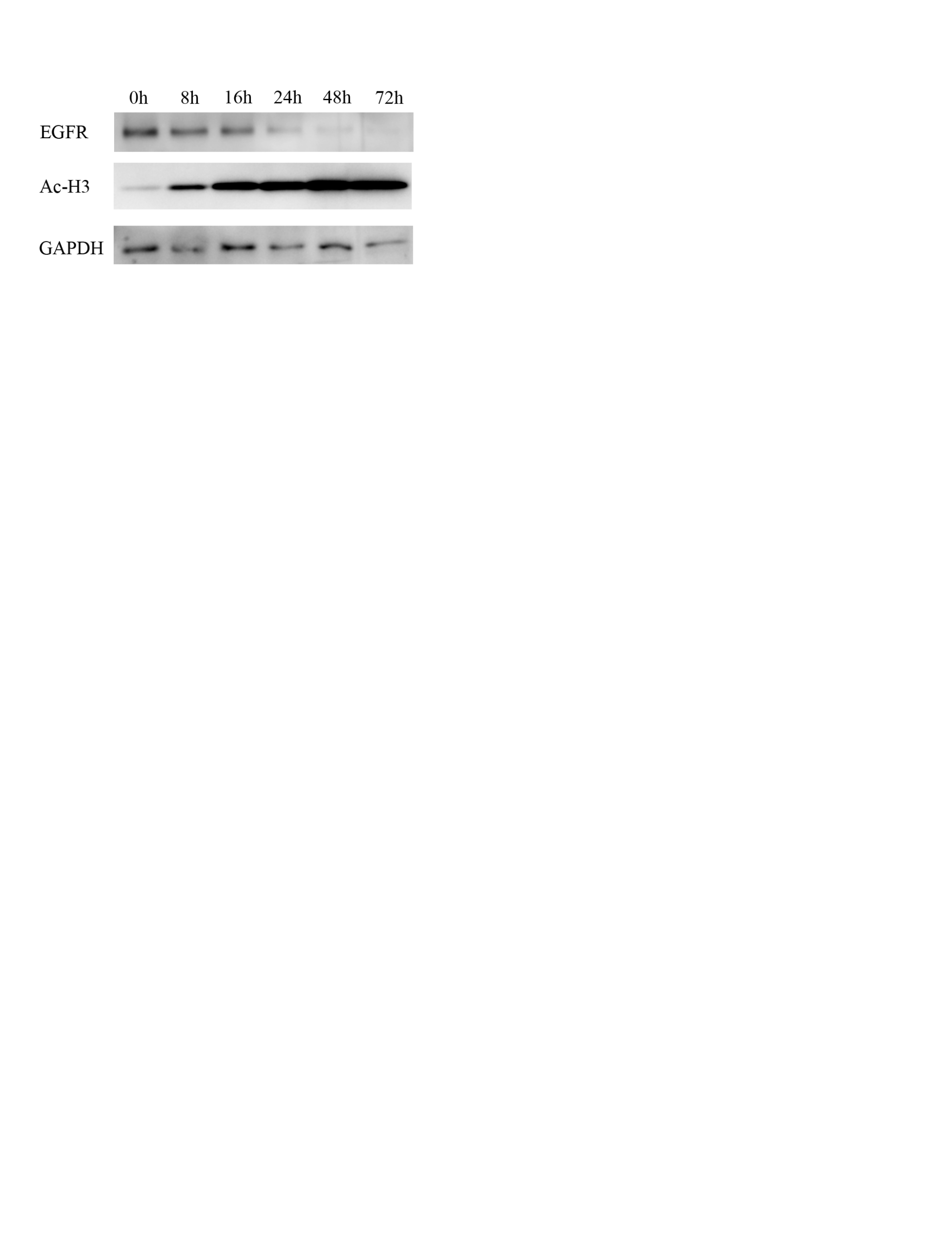


**Figure S13.** The EGFR degradation in H1975-OR treated with GM-protac (3 μM) at 0 h, 8 h, 16 h, 24 h, 48 h, 72 h.


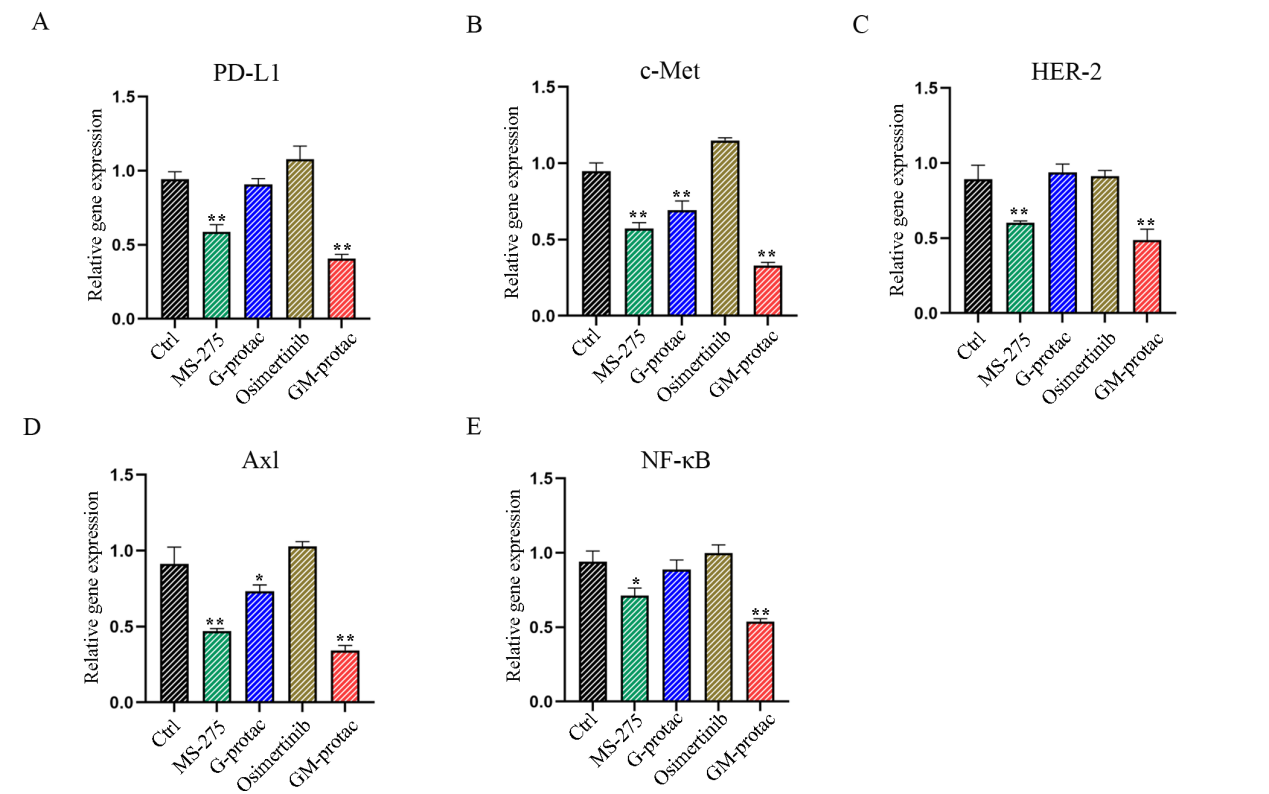


**Figure S14.** The mRNA levels of different proteins regulated by different drugs.The mRNA levels of cancer-related genes in H1975-OR cells incubated with treated with MS275 (1 μM), osimertinib (5 μM), G-protac (6 μM), GM-protac (3 μM) for 48 hours. Fold changes of mRNA levels were analyzed by quantitative PCR. Error bars represent SEMs of at least three independent measurements. .*, P < 0.05, **, P < 0.01 vs Ctrl.


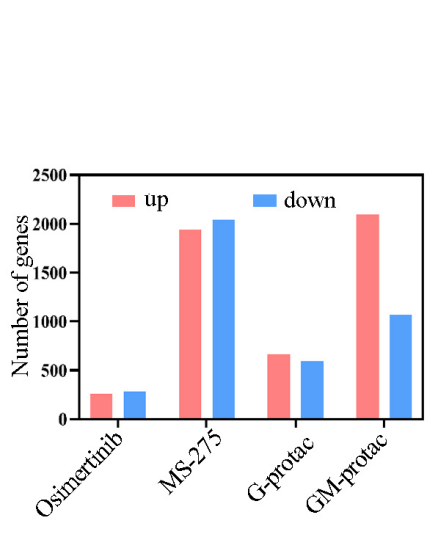


Figure S15. The upregulated and downregulated genes in microarray analysis treated with MS275(1 μM), osimertinib (5 μM), G-protac (6 μM), GM-protac (3 μM) in H1975-OR cells.


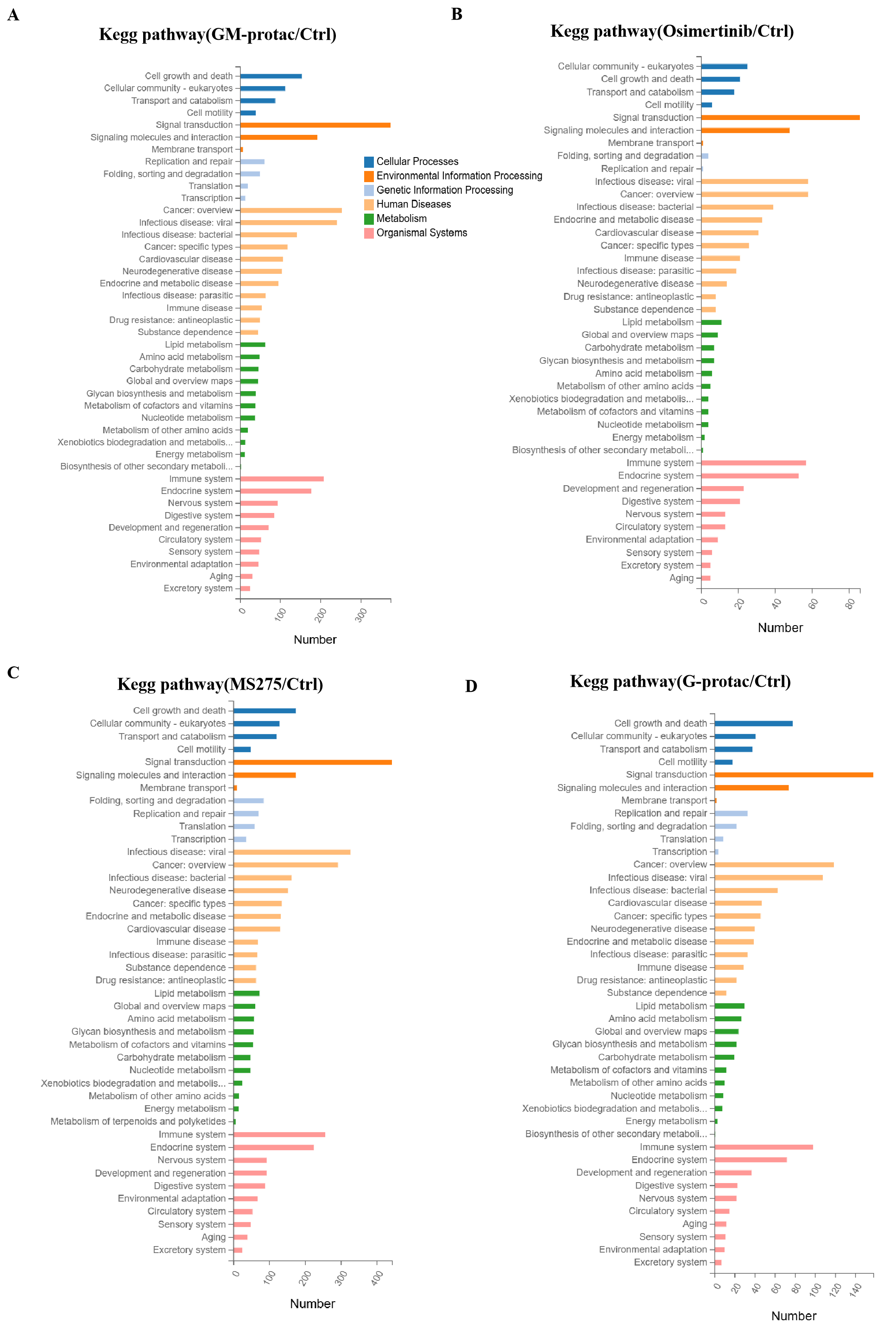


**Figure S16.** KEGG classification revealed different signaling pathways influenced by GM-protac, G-protac, Osimertinib or MS275 in H1975-OR cells. (A) GM-protac/Ctrl, (B) Osimertinib /Ctrl, (C) MS275/Ctrl, (D) G-protac/Ctrl.


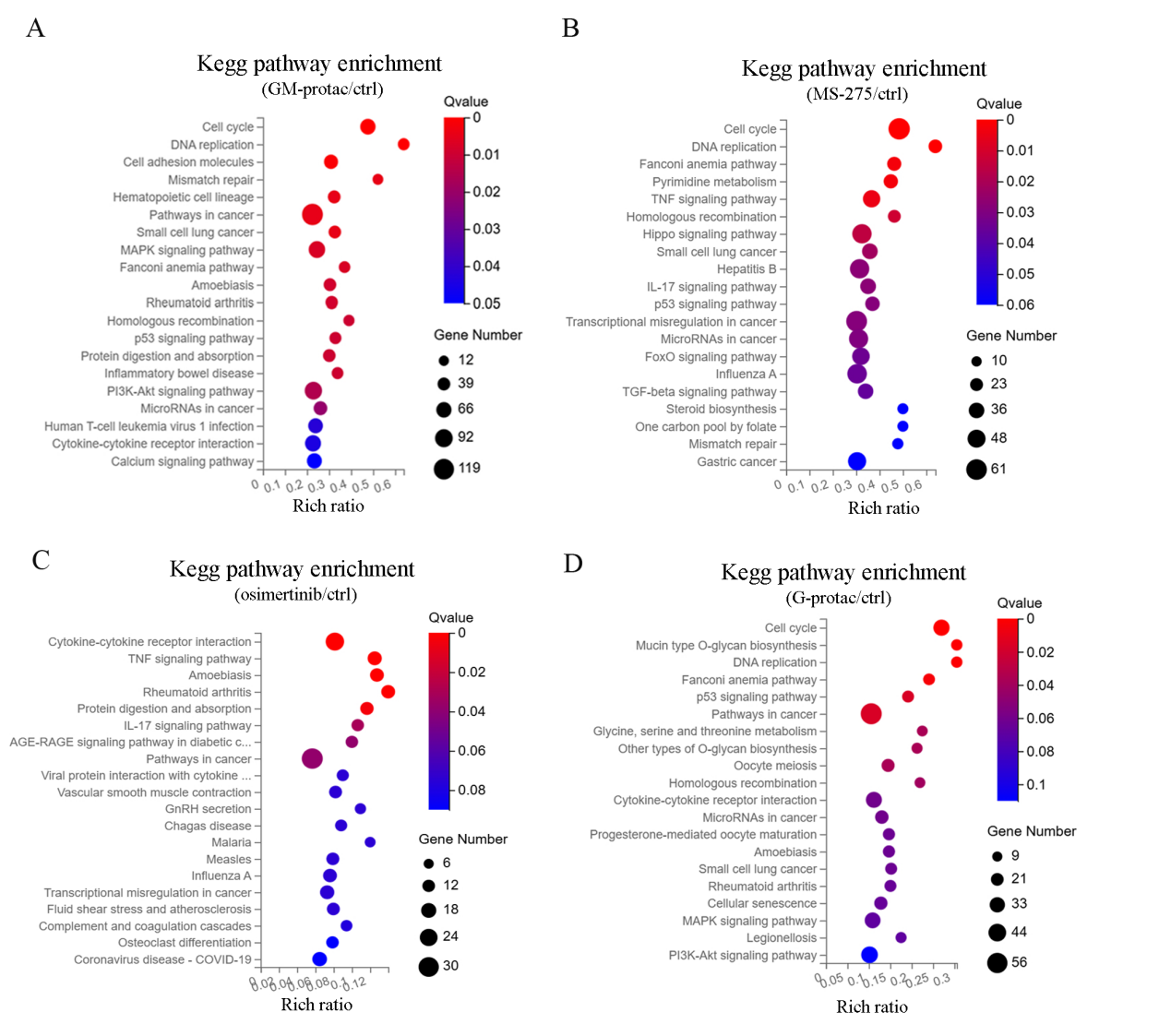


**Figure S17.** Kegg pathway enrichment revealed different signaling pathways influenced by GM-protac (A), MS275 (B), Osimertinib (C), and G-protac (D).


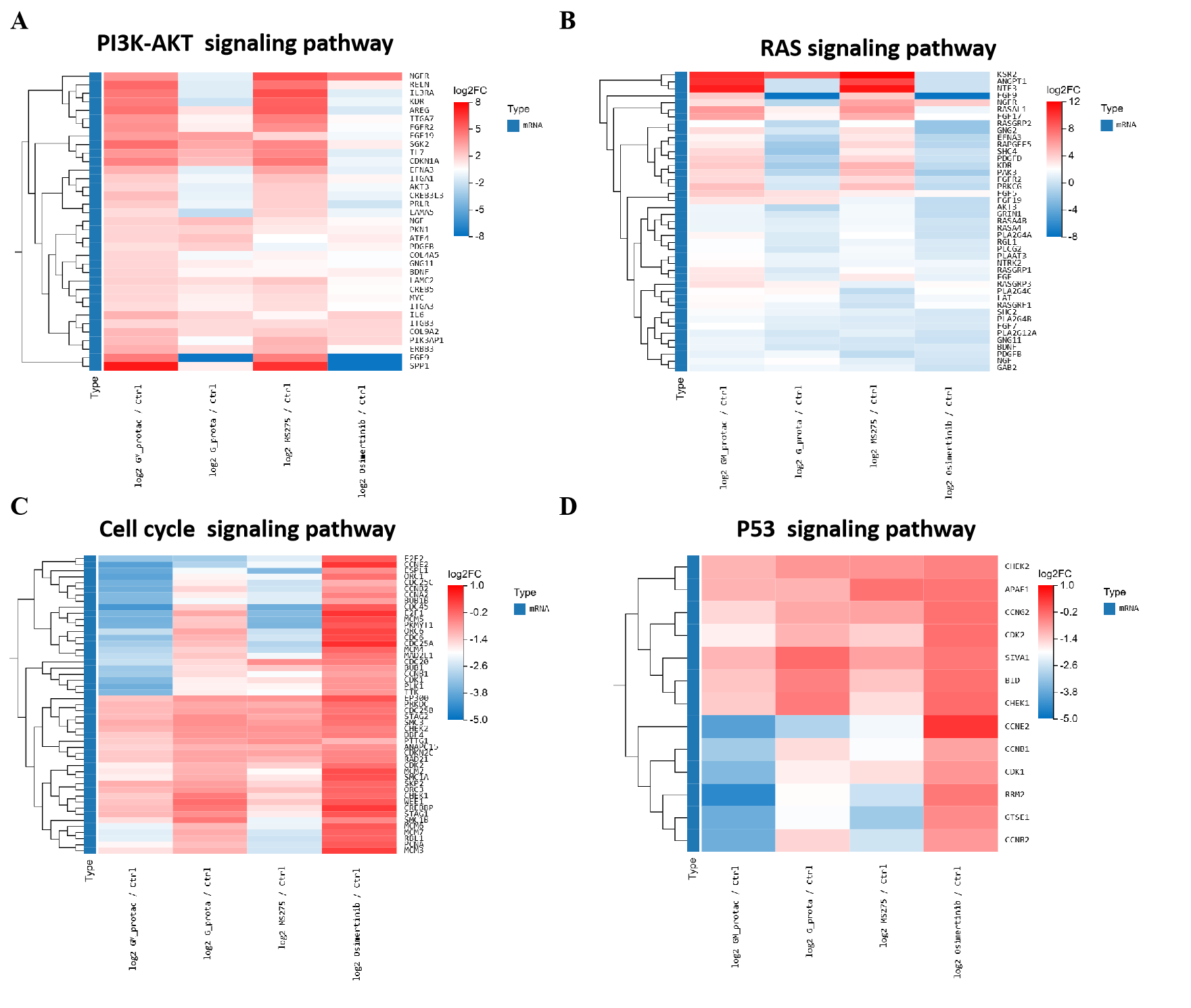


**Figure S18.** Changes in the expression of genes associated with different signaling pathways influenced by GM-protac, G-protac, Osimertinib or MS275 in H1975-OR cells. (A)PI3K-AKT signaling pathway, (B)RAS signaling pathway, (C)Cell cycle signaling pathway, (D)P53 signaling pathway.


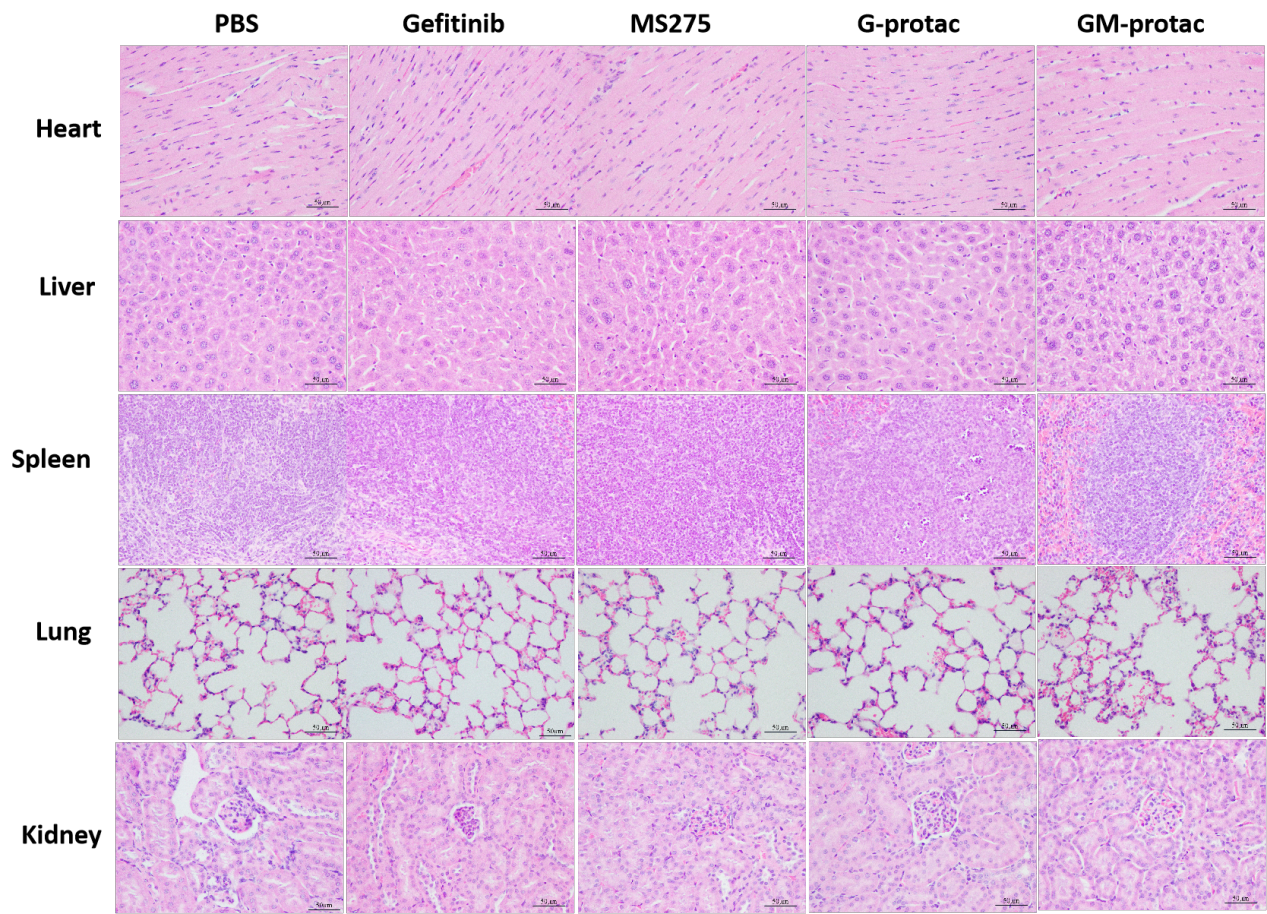


**Figure S19.** H&E staining of different organ treated with different drugs in H1975 tumor bearing mice models.


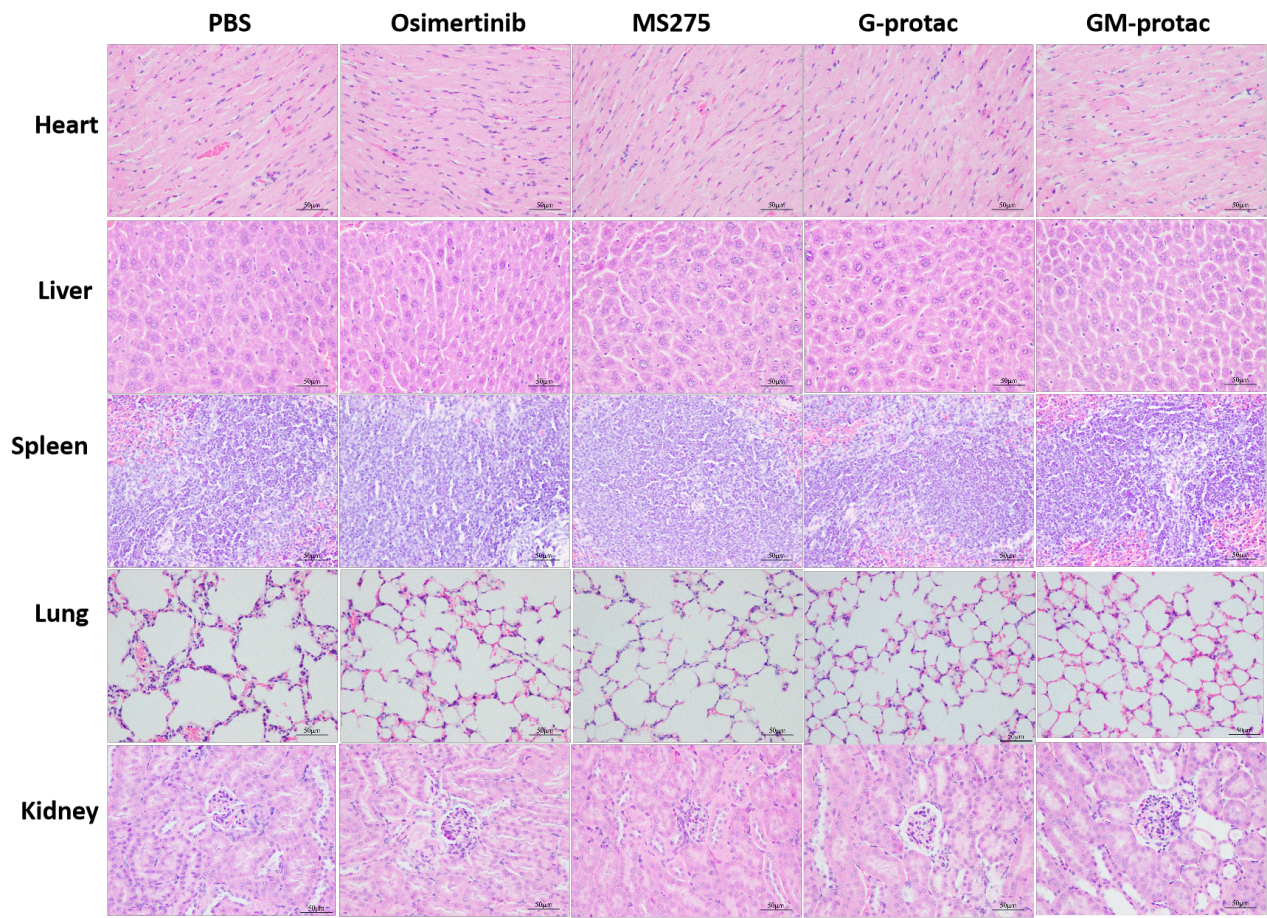


**Figure S20.** H&E staining of different organ treated with different drugs in H1975-OR tumor bearing mice models.

**References**

[1] Song C, Jiao Z, Hou Z, Wang R, Lian C, Xing Y, Luo Q, et al. *J Am Chem Soc* **2023**, 145, 21860-21870.
